# Strategies to Enhance COVID-19 Vaccine Uptake among Prioritized Groups, Uganda–Lessons Learned and Recommendations for Future Pandemics

**DOI:** 10.3201/eid3007.231001

**Published:** 2024-07

**Authors:** Daniel Kiiza, Judith Nanyondo Semanda, Boneventure Brian Kawere, Claire Ajore, Christopher Kaliisa Wasswa, Andrew Kwiringira, Emmanuel Tumukugize, Joel Sserubidde, Nashiba Namyalo, Ronald Baker Wadria, Peter Mukiibi, Julie Kasule, Ivan Chemos, Acham Winfred Ruth, Ritah Atugonza, Flora Banage, Yvette Wibabara, Immaculate Ampaire, Alfred Driwale, Waverly Vosburgh, Lisa Nelson, Mohammed Lamorde, Amy Boore

**Affiliations:** Makerere University Infectious Diseases Institute, Kampala, Uganda (D. Kiiza, J. Nanyondo Semanda, B.B. Kawere, C. Ajore, C.K. Wasswa, A. Kwiringira, N. Namyalo, R. Baker Wadria, P. Mukiibi, M. Lamorde);; Mildmay Uganda, Kampala (E. Tumukugize, J. Sserubidde);; Centers for Disease Control and Prevention, Atlanta, Georgia, USA (J. Kasule, F. Banage, Y. Wibabara, W. Vosburgh, L. Nelson, A. Boore);; Rakai Health Sciences Program, Kalisizo, Uganda (I. Chemos);; The AIDS Support Organization, Kampala (A.W. Ruth);; Ministry of Health, Kampala (R. Atugonza, I. Ampaire, A. Driwale)

**Keywords:** COVID-19, SARS-CoV-2, viruses, respiratory infections, zoonoses, vaccine-preventable diseases, vaccines, high-priority populations, Uganda

## Abstract

COVID-19 vaccination was launched in March 2021 in Uganda and initially prioritized persons >50 years of age, persons with underlying conditions, healthcare workers, teachers, and security forces. However, uptake remained low 5 months after the program launch. Makerere University’s Infectious Diseases Institute supported Uganda’s Ministry of Health in optimizing COVID-19 vaccination uptake models by using point-of-care, place of worship, and place of work engagement and the Social Assistance Grant for Empowerment model in 47 of 135 districts in Uganda, where we trained influencers to support mobilization for vaccination outreach under each model. During July–December, vaccination rates increased significantly in targeted regions, from 92% to 130% for healthcare workers, 40% to 90% for teachers, 25% to 33% for security personnel, 6% to 15% for persons >50 years of age, and 6% to 11% for persons with underlying conditions. Our approach could be adopted in other targeted vaccination campaigns for future pandemics.

Globally, the COVID-19 pandemic resulted in >650 million infections and 6.5 million deaths during March 2020–December 2022 (*1*,*2*). Uganda recorded its first case of SARS-CoV-2 infection on March 21, 2020; as of January 20, 2023, Uganda had reported >170,000 cases and 3,630 deaths (*2*–*4*).

SARS-CoV-2 infections can have various clinical manifestations, ranging from asymptomatic infection to mild-to-severe and critical respiratory illnesses requiring hospitalization. Vulnerable populations, such as healthcare workers and persons with underlying conditions, immune dysfunction, or advanced age, are at increased risk for COVID-19, progression to severe disease, and death (*5*–*7*).

COVID-19 vaccination is critical in reducing severe disease and death in vulnerable populations while protecting health systems and enabling the relaxation of public health measures (*8**–**11*). More important, rapidly vaccinating high-priority groups is crucial for mitigating the effect of the pandemic, similar to emergency immunization response strategies applied during outbreaks of other vaccine-preventable disease (*12*).

In March 2021, the Ministry of Health in Uganda launched the National COVID-19 Vaccine Deployment Plan. Phase 1 targeted priority populations for vaccination, including healthcare workers, other essential workers (including security personnel and teachers), persons >50 years of age, and patients with underlying chronic medical conditions (*13*,*14*). This approach considered the heightened global demand for COVID-19 vaccine limiting availability and driving vaccine inequity to lower-income countries at the time and the disproportionate occurrence of severe COVID-19 disease and death in vulnerable groups in Uganda (*15*). As such, a phased vaccine deployment approach prioritizing vulnerable groups was crucial to maximize the public health gains from the rollout. However, 5 months into phase 1 rollout, COVID-19 vaccination coverage in those groups remained low (*16*,*17*).

To address this shortfall, the US Centers for Disease Control and Prevention (CDC) and the Infectious Diseases Institute (IDI) at Makerere University in Uganda implemented a project to support the Uganda Ministry of Health (MoH) through the Uganda National Expanded Program for Immunization (UNEPI) in accelerating vaccination uptake in high-priority groups. We describe strategies designed and deployed to enhance the COVID-19 vaccination uptake among prioritized groups.

## Methods

### Project Description Strategy and Context

IDI launched the COVID-19 vaccination project through its Global Health Security Department in July 2021 to increase COVID-19 vaccine uptake among national priority groups through national and subnational implementational support to MoH and UNEPI. The project recruited 3 officers and deployed them at MoH and UNEPI with distinct roles in supporting UNEPI in developing strategies for vaccine advocacy and vaccine safety and technical coordination of all subgranted partners in the regional implementation of vaccine service delivery support. With national-level coordination from the IDI, funding was provided to 4 organizations already receiving funding from the US President’s Emergency Plan for AIDS Relief (PEPFAR) for the HIV Comprehensive Care Program through CDC. This funding was designated to support project implementation in 5 regions across 47 districts and 5 cities. The funded implementing partners and areas of coverage included IDI, reaching 12 districts and 1 city in the West Nile Region and the 1 district and 2 cities of Kampala (metropolitan district of Kampala) and Entebbe (metropolitan district of Wakiso); the Rakai Health Sciences Program, reaching 12 districts and 1 city in South Central (Southern Buganda) Region; the AIDS Support Organization (TASO), covering 10 districts of Teso Region and 4 districts of Southern Karamoja Region; and Mildmay Uganda, covering 8 districts in the North Central (Northern Buganda) Region.

**Figure 1:**
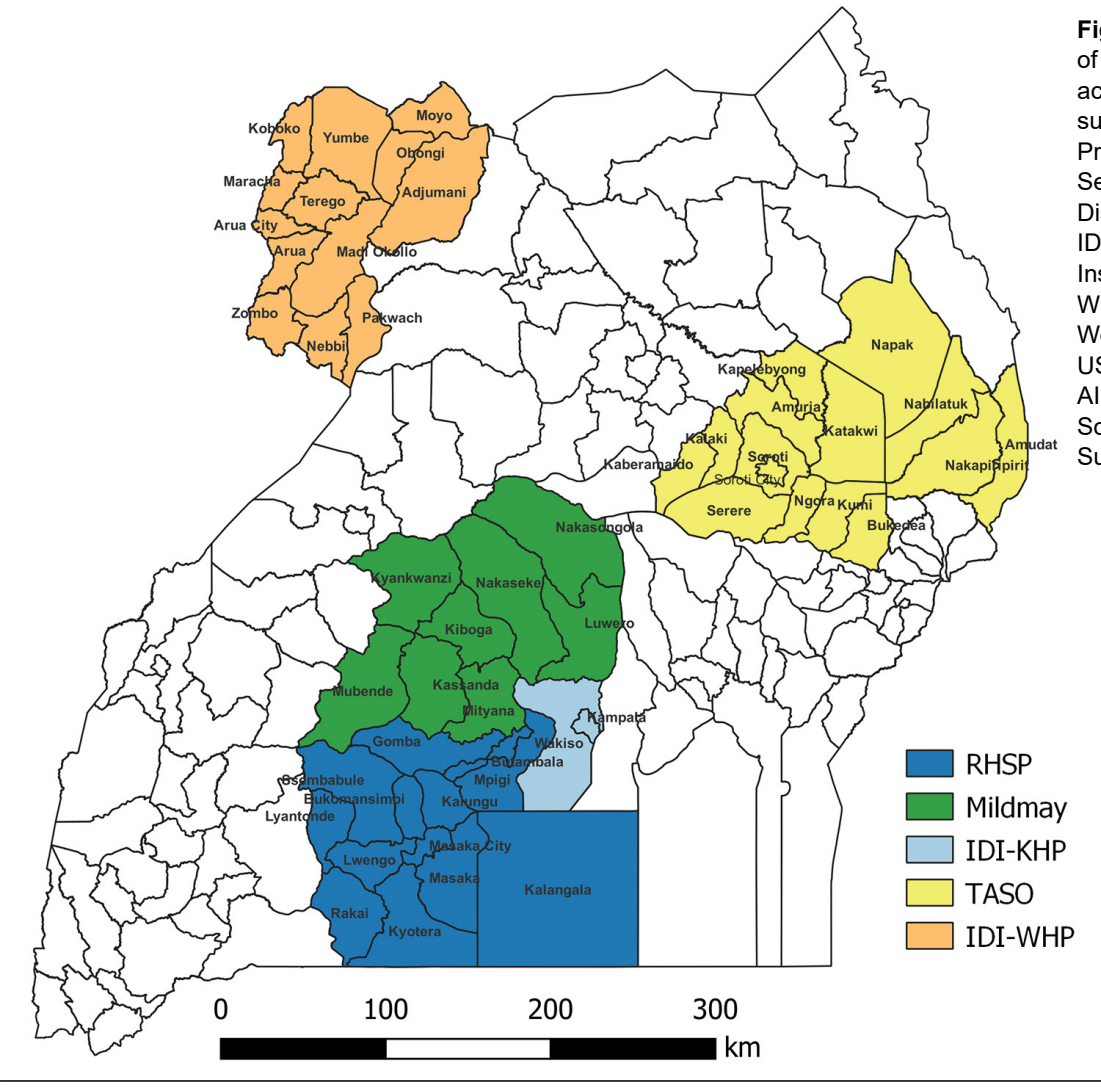
Regional implementation of COVID-19 vaccination project across 47 districts and 5 cities supported by the CDC–PEPFAR Program, Uganda, July 2021–September 2022. CDC, Centers for Diseases Control and Prevention; IDI-KHP; Infectious Diseases Institute–Kampala HIV Project; IDI-WHP, Infectious Diseases Institute–West Nile HIV Project; PEPFAR, US President’s Emergency Plan for AIDS Relief; RHSP, Rakai Health Sciences Program; TASO, The AIDS Support Organization.

Each implementing partner recruited >4 personnel, including a regional coordinator and data officers. The goal was to layer project implementation into the broader PEPFAR HIV Comprehensive Care Program, which employs a cluster approach with, on average, 4 clusters in each geographic region. MoH technical experts used the adapted version of the World Health Organization (WHO) online COVID Vaccination Training for Health Workers (https://openwho.org/courses/covid-19-vaccination-healthworkers-en) to train all regional implementing officers to ensure adequate support of government vaccination activities in the 47 supported districts and 5 cities. Furthermore, this effort was supplemented with supportive supervision visits from the national team to reinforce appropriate project implementation, link stakeholder groups to implementing partners, and roll out the developed vaccination models (Figure 1).

The project aimed to accelerate COVID-19 vaccination among priority populations by developing evidence-based strategies and models for vaccination uptake. We used guidance from the WHO Behavioral and Social Drivers of the Vaccination Framework (*18*) to inform our approach with interventions that included community engagement, dialogue-based approaches, interpersonal advocacy efforts, and targeted vaccination outreach.

### Stakeholder Mapping and Engagement

In partnership with UNEPI, project technical personnel provided national-level assistance in mapping stakeholders associated with priority populations for phase 1 vaccination, engaging them through in-person and virtual teleconference meetings. We discussed the challenges of vaccination uptake among priority populations and brainstormed strategies to address these challenges, highlighting the stakeholders’ role. 

### COVID-19 Vaccination Uptake Models

The strategies described informed the development of COVID-19 vaccination uptake models to accelerate vaccination among priority populations. As part of national-level technical support to the MoH and UNEPI, models were conceptualized and subsequently rolled out at the subnational level, collaborating with subgranted implementing partners in respective districts.

#### Point-of-Care Vaccination Model

Under this model, we selected high-volume health facilities with specialized clinics for chronic conditions such as HIV, diabetes, and cardiovascular disease. We identified healthcare workers and key influencers among patients in these clinics and trained them to be vaccination champions to support interpersonal mobilization for COVID-19 vaccination within the clinics. We distributed information, education, and communication (IEC) materials to assist with mobilization efforts. Furthermore, we built healthcare workers’ capacity to screen and line-list eligible patients for COVID-19 vaccination. District vaccination teams were then linked to work closely with the vaccination champions to conduct targeted outreach on prespecified clinic days.

#### Place of Work Model

We identified corporate entities and their leaders and used virtual platforms to train the leaders and their staff members to be vaccination champions. During training sessions, we provided essential IEC materials and e-posters to support the interpersonal mobilization of leaders and staff members’ family members and colleagues for COVID-19 vaccination. We then linked district vaccination teams to the corporate entities’ management to conduct vaccination outreaches at the corporate headquarters or preferred locations, such as playgrounds and recreation centers.

#### Place of Worship Model

We identified places of worship and their leaders, including key influencers within the congregations, and trained them to be vaccination champions. We provided these persons with IEC materials to support interpersonal mobilization for vaccination. This effort was followed by linking vaccination teams to these places of worship to conduct outreach vaccination outreach after prayer.

#### Social Assistance Grant for Empowerment (SAGE) Model

The government of Uganda operates a social protection program offering quarterly stipends to persons >80 years of age. Those payments are administered at the subcounty level under district Community Development Officers’ (CDO) oversight. We conducted training sessions for the CDOs and influential persons >80 years of age, appointing them to be vaccination champions to spearhead interpersonal mobilization efforts. Subsequently, we connected district vaccination teams with the CDOs and vaccination champions to conduct targeted vaccination outreach near payment sites.

### Development of the COVID-19 Vaccination Champions Toolkit

We trained the vaccination champions and key influencers identified for each model described by using the COVID-19 Vaccination Champions Toolkit (Appendix) for conducting interpersonal and social mobilization of their communities for vaccination. IDI supported MoH in developing the Vaccination Champion’s toolkit, which consists of 3 modules. Module 1 provides an overview of COVID-19 basics, including information on transmission, prevention strategies, and identifying the most vulnerable persons requiring vaccination. Module 2, focused on vaccines, addresses safety concerns and guidelines for reporting adverse effects after immunization. Module 3 focuses on communication strategies related to vaccination, incorporating key insights from WHO’s Behavioral and Social Drivers of the Vaccination Framework (*18*).

We developed the toolkit by adapting existing UNEPI COVID-19 vaccination training materials for healthcare workers for lay audiences, coopting WHO explainers on COVID-19 and vaccines (*19*), and tailoring the San Francisco Public Health Department and the University of California–San Francisco Vaccine Ambassador training program to the situation of Uganda (*20*). We convened a 1-day workshop on October 8, 2021, to adapt the drafted toolkit with key inputs from the MoH and UNEPI Advocacy and Risk Communication Department and a patient advocacy group, Community Health Advocacy and Information Network.

We used the mobile communication application WhatsApp as a virtual collaborative platform for sharing electronic IEC materials and updates on vaccine availability and vaccination locations with trained vaccination champions. In addition, we used the platform to counter the evolving misinformation and disinformation surrounding the COVID-19 vaccination program.

### Data Management and Ethics Considerations

COVID-19 vaccination uptake was the outcome variable of interest, which we defined as the number of persons vaccinated with a certain dose of the vaccine in a certain period expressed as the proportion of a target population (*21*). We extracted aggregate COVID-19 vaccination uptake data, categorized by prioritized groups, from the District Health Information System 2.0 database. We transferred those data, devoid of unique identifier information, to a computer with restricted access. Subsequently, we used the data during March–December 2021 to generate vaccination uptake trend curves for prioritized groups in the PEPFAR-supported districts. This intervention was implemented in response to a public health emergency after receiving authorization from MoH’s Office of the Director General of Health Services.

## Results

### Stakeholder Mapping and Engagement

We engaged 23/30 (77%) of the mapped stakeholders’ groups in 6 in-person and 7 virtual meeting sessions. The initial meetings with stakeholders were mainly physical, intended to establish rapport, update stakeholders on the challenges of vaccination in the priority population, and discuss the role they can play. A total of 44 leaders of stakeholder groups participated in the physical meetings and the subsequent virtual meeting that involved training 1,333 members to be vaccination champions. Three of 6 of the initial engagements with the stakeholder leadership blended physical and virtual meetings.

**Figure 2:**
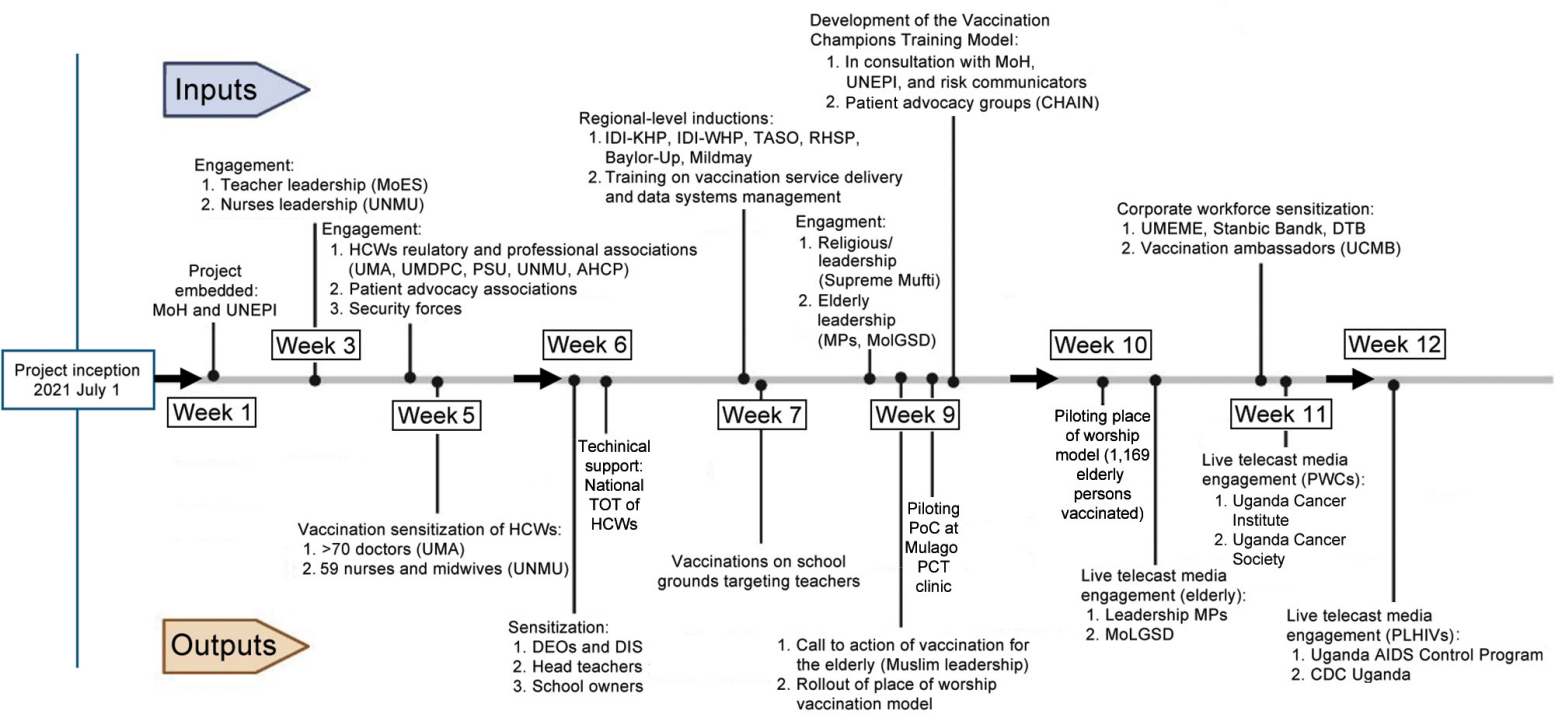
Timeline series of engagements and their respective outputs (starting July 1, 2021) within the first 12 weeks of the COVID-19 vaccination project, Uganda, July–September 2021. ACP, AIDS Control Program; AHPC, Allied Health Practitioners Council; CDC, US Centers for Disease Control and Prevention; CHAIN, Community Health and Information Network; DEOs, District Education Officers; DIS, District Inspector of School; DTB, Diamond Trust Bank; HCWs, healthcare workers; IDI-KHP, Infectious Diseases Institute–Kampala HIV Project; IDI-WHP, Infectious Diseases Institute–West Nile HIV Project; MoES, Ministry of Education and Sports; MoH, Ministry of Health; MoLGSD, Ministry of Gender, Labour, and Social Development; MPs, Members of Parliament; PCT, HIV Prevention Care and Treatment; PoC, point-of-care; PSU, Pharmacy Council, Pharmaceutical Society of Uganda; PWCs, persons with underlying conditions; RHSP, Rakai Health Sciences Program; TASO, The AIDS Support Organization; ToT, training of trainers; UCI, Uganda Cancer Institute; UCMB, Uganda Catholic Medical Bureau; UDA, Uganda Diabetes Association; UHI, Uganda Heart Institute; UMA, Uganda Medical Association; UMDPC, Uganda Medical and Dental Practitioners Council; UNEPI, Uganda National Expanded Program on Immunization; UNMC, Uganda Nurses and Midwives Councils; UNMU, Uganda Nurses and Midwives Union.

Some of the engagements with the stakeholders lead to key outputs, such as mobilizing members for training as vaccination champions, piloting the models, engaging media to call to action priority populations for vaccination, and conducting targeted outreach of stakeholder members. Specifically, 3 stakeholders designated places of worship for targeted vaccination outreach campaigns (Watoto Ministries, Kakande Ministries, and Gadhafi Mosque). This process resulted in vaccinations from direct mobilization support from the stakeholders after the engagement with stakeholders targeting the key priority populations that are part of the stakeholder membership (Table 1; Figure 2). 

**Table 1:** Engagement of stakeholders linked to the priority populations for COVID-19 vaccination, Uganda, July–September 2021

Category	Stakeholders engaged	Physical meeting	Virtual meetings
Healthcare workers	Uganda Medical and Dental Practitioners Council; Uganda Medical Association	9 members, including Uganda Medical and Dental Practitioners Council Registrar and Uganda Medical Association President	70 doctors; 380 infection prevention and control specialists
	Uganda Nurses and Midwives Councils; Uganda Nurses and Midwives Union (UNMU)	UNMU President and Uganda Nurses and Midwives Councils Registrar	56 national executive committee personnel to the UNMU and chairpersons countrywide
	Pharmacy Council; Pharmaceutical Society of Uganda; Allied Health Practitioners Council	15 members of the pharmacy council and Pharmaceutical Society of Uganda engaged in both virtual and physical session	80 pharmacists sensitized on COVID-19 vaccination
Teachers	Uganda National Teachers Union; Ministry of Education and Sports; Ministry of Local Government	Chairperson, commissioner, and permanent secretary	131 district education officers, 500 private school owners, private teachers association, faith-based schools
Security personnel	Uganda Peoples’ Defense Forces; Uganda Police Force; Uganda Prison Services	5 personnel concerned with health and medical matters	
Persons >50 years of age	Ministry of Gender, Labour, and Social Development; members of parliament for persons >50 years of age; faith-based organizations	Supreme Mufti, church leadership, 3 members of parliament, and the state minister	47 members
Persons with underlying conditions	Community Health and Information Network; Uganda Diabetes Association; Uganda Heart Institute; Uganda Cancer Institute; Uganda AIDS Control Program; corporate entities (Stanbic Bank, UMEME Limited, Diamond Trust Bank)	17 members	

### COVID-19 Vaccination Uptake by Models

We assessed COVID-19 vaccination by vaccination model (Table 2). During the implementation period of September–December 2021, a total of 75,098 vulnerable priority persons were vaccinated through activities and outreach based on the models. All 4 models were piloted in Kampala, where all stakeholder engagements occurred and were hosted in the IDI and Kampala HIV Project operational region. Those models were subsequently rolled out to other implementing regions. We assessed the percentage of overall COVID-19 vaccinations attributable to each model: point-of-care, 11%; place of worship, 38%; place of work, 8%; and SAGE, 43%.

**Table 2:** Contribution of vaccination models targeting priority groups for COVID-19 vaccination uptake in CDC–PEPFAR supported districts, Uganda, September–December 2021*

Vaccination model	CDC PEPFAR implementing partners					Total no. (%)
	IDI-KHP	IDI-WHP	Mildmay Uganda	TASO	RHSP	
Point-of-care	4,096	427	0	787	3,020	8,330 (11)
Place of worship	27,284	330	0	316	570	28,500 (38)
Place of work	2,711	1,000	0	1891	200	5,802 (8)
SAGE	694	13,731	1,808	16,233	0	32,466 (43)
Total	34,785	15,488	1,808	19,227	3,790	75,098 (100)

*CDC, Centers for Disease Control and Prevention; IDI-KHP, Infectious Diseases Institute–Kampala HIV Project; IDI-WHP, Infectious Diseases Institute–West Nile HIV Project; PEPFAR, US President’s Emergency Plan for AIDS Relief; RHSP, Rakai Health Sciences Program; SAGE, Social Assistance Grant for Empowerment; TASO, The AIDS Support Organization.

Phase 1 of the National COVID-19 Vaccine Deployment Plan aimed to vaccinate 4.8 million priority persons (150,000 healthcare workers, 550,000 teachers, 250,000 security personnel, 3,348,5000 persons >50 years of age, and 500,000 persons with underlying conditions). In July 2021, 5 months into the national COVID-19 vaccination roll-out in Uganda, national-level vaccination uptake among those groups (by receipt of first dose) stood at 94,684/150,000 (63.1%) for healthcare workers, 158,406/550,000 (29%) for teachers, 142,509/250,000 (57%) for security personnel, 276,736/ 3,384,000 (8%) for persons >50 years of age, and 25,361/500,000 (5.1%) for persons with underlying conditions. As of November 15, 2021, approximately 4 months into the implementation of project activities from inception in July 2021, vaccination uptake improved to 140,635 (93.8%) for healthcare workers, 376,555 (68.595%) for teachers, 161,491 (64.6%) for security personnel, 511,142 (15.3%) for persons >50 years of age, and 40,443 (8.1%) for persons with underlying conditions.

For programmatic tracking, the CDC–PEPFAR supported districts were assigned 72,225 healthcare workers, 264,825 teachers, 120,375 security personnel, 1,612,304 persons >50 years of age, and 240,750 persons with underlying conditions as target contributions to the overall national target for priority populations for phase 1. Three months after initiation of project activities in the CDC–PEPFAR supported districts, vaccination uptake improved from 66,561/72,225 (92%) to 93,889/72,225 (130%) for healthcare workers, 105,310/264,825 (40%) to 238,791/264,825 (90%) for teachers, 29,808/120,375 (25%) to 40,041/120,375 (33%) for security personnel, 100,788/1,612,304 (6%) to 235,439,1,612,304 (15%) for persons >50 years of age, and 14,878/240,750 (6%) to 26,134/240,750 (11%) for persons with underlying conditions.

**Figure 3:**
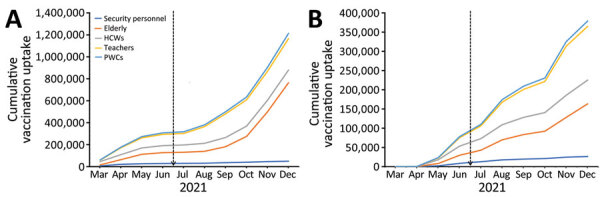
COVID-19 vaccination coverage among priority populations, by receipt of first (A) and second (B) dose, showing project inception date (vertical dashed line) and vaccine uptake trends among priority populations, Uganda, March–December 2021. HCWs, healthcare workers; PWCs, persons with underlying conditions.

By the close of 2021, a total of 115,737/72,225 (160%) healthcare workers, 285,369/264,825 (108%) teachers, 49,773/120,375 (41%) security personnel, 713,776/1,612,304 (44%) persons >50 years of age, and 47,892/240,750 (20%) persons with underlying conditions were vaccinated in CDC–PEPFAR supported districts. In terms of the percentage of vaccinations nationwide per target group, those regions contributed 115,737/153,673 (75%) of vaccinations among healthcare workers, 285,369/403,184 (71%) of vaccinations among teachers, 49,773/161,491 (31%) of vaccinations among security personnel, 713,776/1,314,706 (54%) of vaccinations among persons >50 years of age, and 47,892/47,555 (101%) of vaccinations among persons with underlying conditions at the close of 2021 (Figure 3).

## Discussion

Targeting priority populations for COVID-19 vaccination has been challenging globally, and countries in sub-Saharan Africa, including Uganda, are no exception (*22*–*25*). Despite government efforts to ensure COVID-19 vaccines are available to populations, practical issues, perceptions, and social processes within vulnerable target groups present access challenges that hinder achieving high vaccination coverage in these populations. Our study elucidates strategies for effectively targeting priority populations for COVID-19 vaccinations. We offer methodologic insights that are transferrable for the effective rollout of vaccination to future pandemics and outbreaks of other vaccine-preventable diseases with identifiable at-risk populations.

This project has demonstrated that stakeholder involvement is essential in planning and targeting vaccination efforts, particularly in the context of COVID-19 vaccination integration into routine healthcare service delivery, to effectively target vulnerable persons and close gaps between government agencies and communities (*26*,*27*). Community-based stakeholders with ties to target populations play a critical role in fostering vaccination-related interpersonal mobilization. Moreover, local influencers can be mobilized to raise COVID-19 vaccination acceptance rates in priority populations as vaccination champions. Such influencers include religious leaders, community leaders, informed patients, and other notable community representatives. The strategies can be adapted for targeted vaccination campaigns to protect vulnerable populations from future pandemics. Studies have demonstrated that the engagement of influencers in developing and implementing vaccination strategies served to reduce misunderstandings and mistrust regarding COVID-19 vaccinations, rekindled community trust and vaccine confidence, and resulted in increased vaccination rates (*28*–*30*).

Using existing public health delivery platforms and community social structures in emergency response efforts can reinforce health systems’ resilience to future pandemics. Our project demonstrates the potential of leveraging existing public health infrastructures, as observed with the involvement of the PEPFAR Comprehensive HIV Care Program and other community-based organizations linked to priority populations, to support COVID-19 vaccination efforts. During the past 2 decades, HIV programs have cultivated community structures and a strong presence that have earned trust within communities toward public health programs. Those established systems can serve as a solid foundation upon which other pandemic prevention preparedness and response efforts, such as COVID-19 vaccination, can build on (*31*–*33*). Such structures can enhance vaccine access and delivery, especially where community involvement is critical for mobilizing vulnerable populations.

This project also has implications for global health security responses, particularly in rapidly deploying medical countermeasures, such as vaccines, as a part of preparedness and response strategies during outbreaks (*33*). Those measures can be optimized to suit specific contexts to effectively deliver a successful rapid vaccination campaign as an emergency response targeting vulnerable populations. A cautious approach should be taken in integrating health security with HIV programming because this process might impede the effective delivery of HIV care services caused by the inevitable competition for resources. A crucial aspect to consider is efficient allocation of resources for each initiative to prevent overburdening either program. Integration efforts should prioritize the streamlining of resource allocations to ensure effectiveness.

We did not set out to independently assess the acceptability and feasibility of implementing various models, leaving room for future exploration. Similarly, evaluating interventions’ effectiveness was not a primary objective, given the urgent public health crisis posed by the COVID-19 pandemic. Consequently, comparison groups were not established because of potential ethical concerns. However, retrospective investigation is now feasible, necessitating an independent evaluation study. Although we used District Health Information System 2.0 data to report COVID-19 vaccination uptake in supported districts, data backlog and completeness were beyond the project’s control. Of note, targets for priority populations were based on estimates from the Uganda Bureau of Statistics and Ministry of Public Service, potentially leading to overperformance caused by underestimation, particularly among healthcare workers, because private sector estimates were not considered.

The vaccination models developed through stakeholder engagement increased COVID-19 vaccine uptake among prioritized groups in supported regions in Uganda. Embracing this approach as part of future pandemic prevention preparedness and response efforts holds promise for enhancing vaccination uptake. Moreover, we highlight the importance of stakeholder engagement in developing models to mobilize priority populations for COVID-19 vaccination, fostering collaboration, and building public confidence in vaccines between government agencies and the communities. Efforts to target persons in high-priority groups should continue to use these models in a tailored approach during the post–COVID-19 era as a critical stabilization and postrecovery strategy for MoH and UNEPI (*34*).

This project demonstrates that, by leveraging the PEPFAR platform, we effectively and expeditiously deployed vaccination, among other emergency public health interventions, by layering health security on earlier global health initiatives in HIV response. Therefore, we recommend that global health security programs consider adopting these strategies to bolster their resilience and effectively support vaccination programs as part of future pandemic prevention preparedness and response efforts. Such proactive measures will strengthen global health security and safeguard populations against emerging threats.

Appendix Additional information about strategies to enhance COVID-19 vaccine uptake among prioritized groups, Uganda.
