# Supplementary material for: Strategies to Enhance COVID-19 Vaccine Uptake among Prioritized Groups, Uganda–Lessons Learned and Recommendations for Future Pandemics
Source: Emerg Infect Dis. 2024 Jul;30(7):1326–34. doi: 10.3201/eid3007.231001 (PMC11210662; doi:10.3201/eid3007.231001)
Supplement: Appendix — Additional information about strategies to enhance COVID-19 vaccine uptake among prioritized groups, Uganda. [file 23-1001-Techapp-s1.pdf]

Article DOI: https://doi.org/10.3201/eid3007.231001

EID cannot ensure accessibility for supplementary materials supplied by authors. Readers who have difficulty accessing supplementary content should contact the authors for assistance.

Strategies to Enhance COVID-19 Vaccine Uptake among Prioritized Groups, Uganda

Appendix

Vaccination Champions: A Training for Community Mobilizers

Shown on the following pages is the COVID-19 Vaccination Champions Toolkit used to conduct interpersonal and social mobilization of communities for vaccination in this study.

Vaccination Champions
A Training for Community Mobilizers

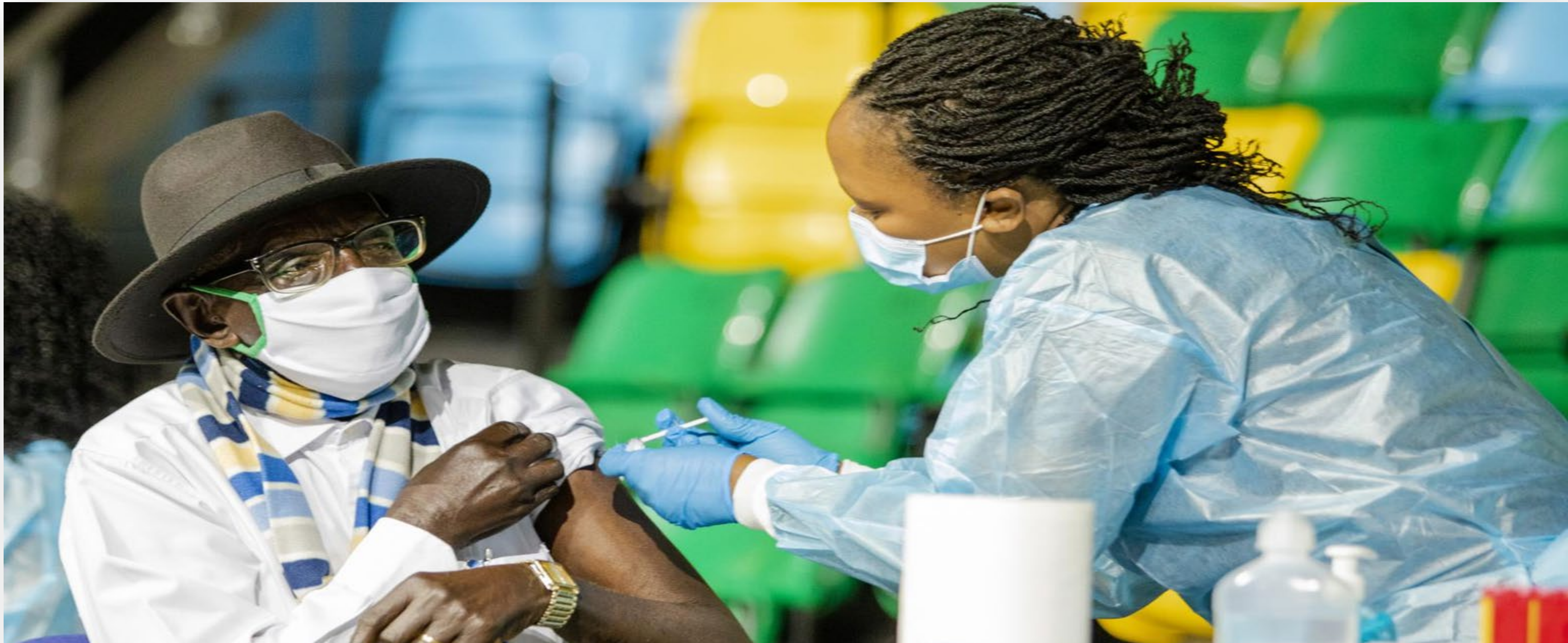

Training modules

Introduction to the course

Part 1: COVID 19

Part 2: Vaccines

Part 3: Vaccine Communication

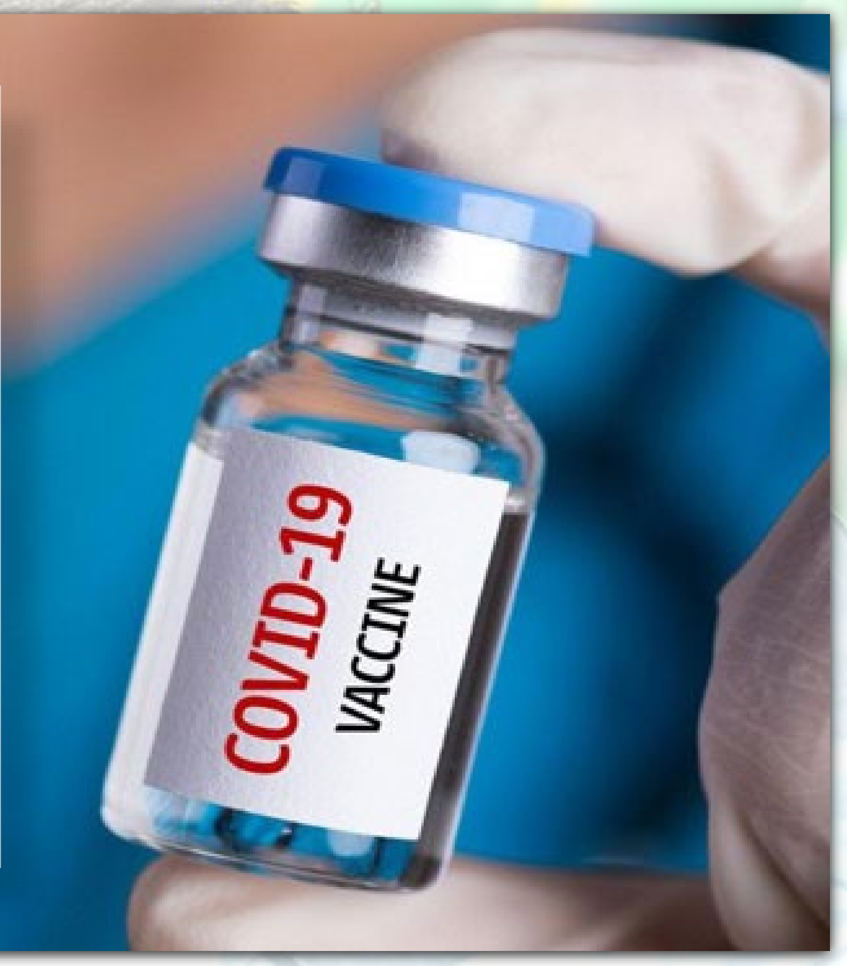

Learning objectives

1

Learn about COVID19, what causes it, how it spreads and how we can prevent it. Recognize priority populations that are targeted for COVID 19 vaccination

2

Know the different types of COVID19 vaccines. Understand how vaccines are monitored for safety

3

Learn how to communicate to people and communities about COVID-19 vaccination

Details of the training

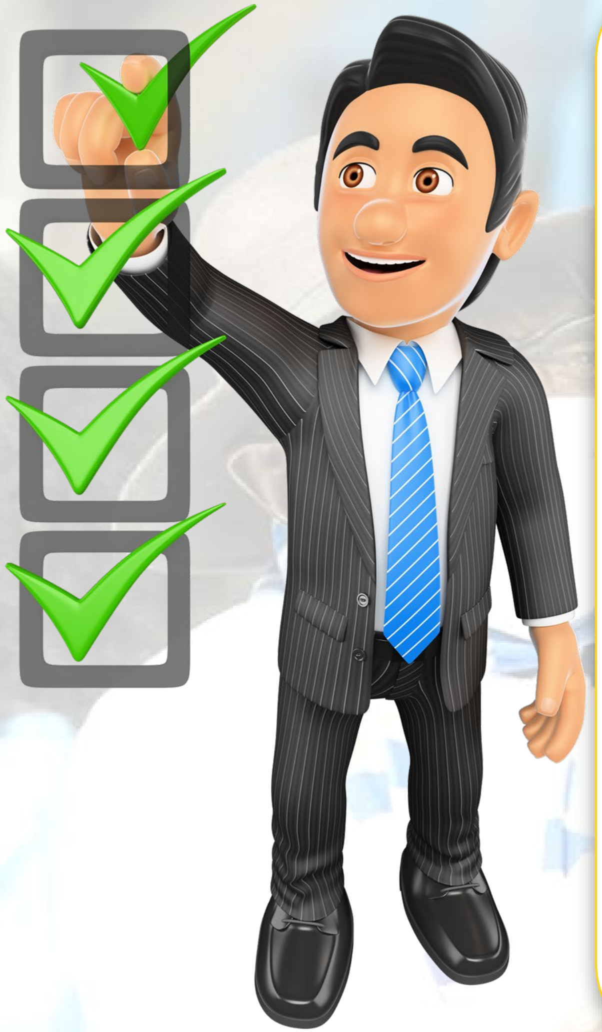

- **Content**: COVID-19 disease, Priority Groups, Vaccines, Safety, Communication
- **Duration**: 90-minutes
- **Target**: District staff, FBO leaders, community leaders, media
- **Train:** Mobilizers
- **Reporting:** Provide activity reports

Definition of vaccination champions

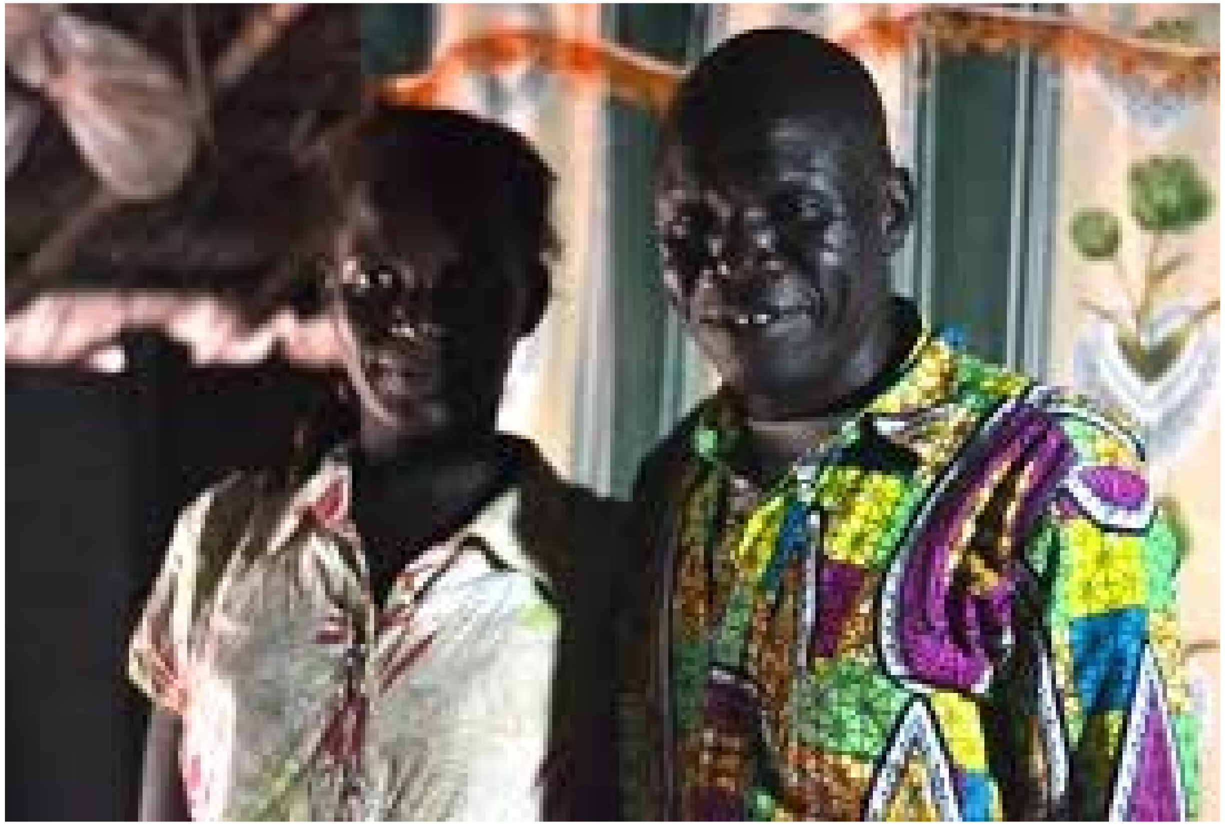

A person who helps someone else overcome a barrier/obstacle to vaccination is a **Vaccination Champion**.

https://svhealthcare.org/news/vaccination-champions

Barriers to vaccination

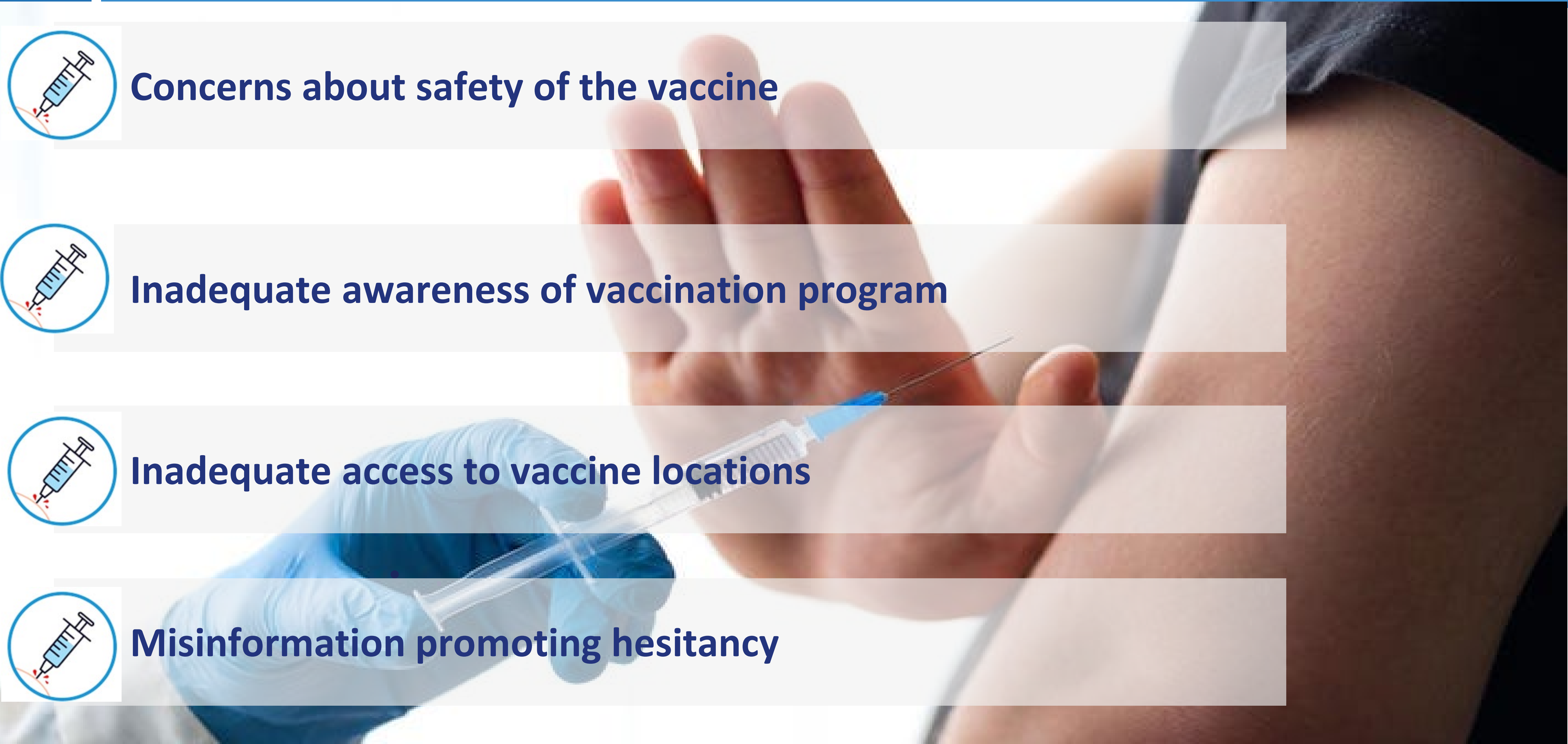

- Concerns about safety of the vaccine
- Inadequate awareness of vaccination program
- Inadequate access to vaccine locations
- Misinformation promoting hesitancy

Example: Vaccine champion in Uganda

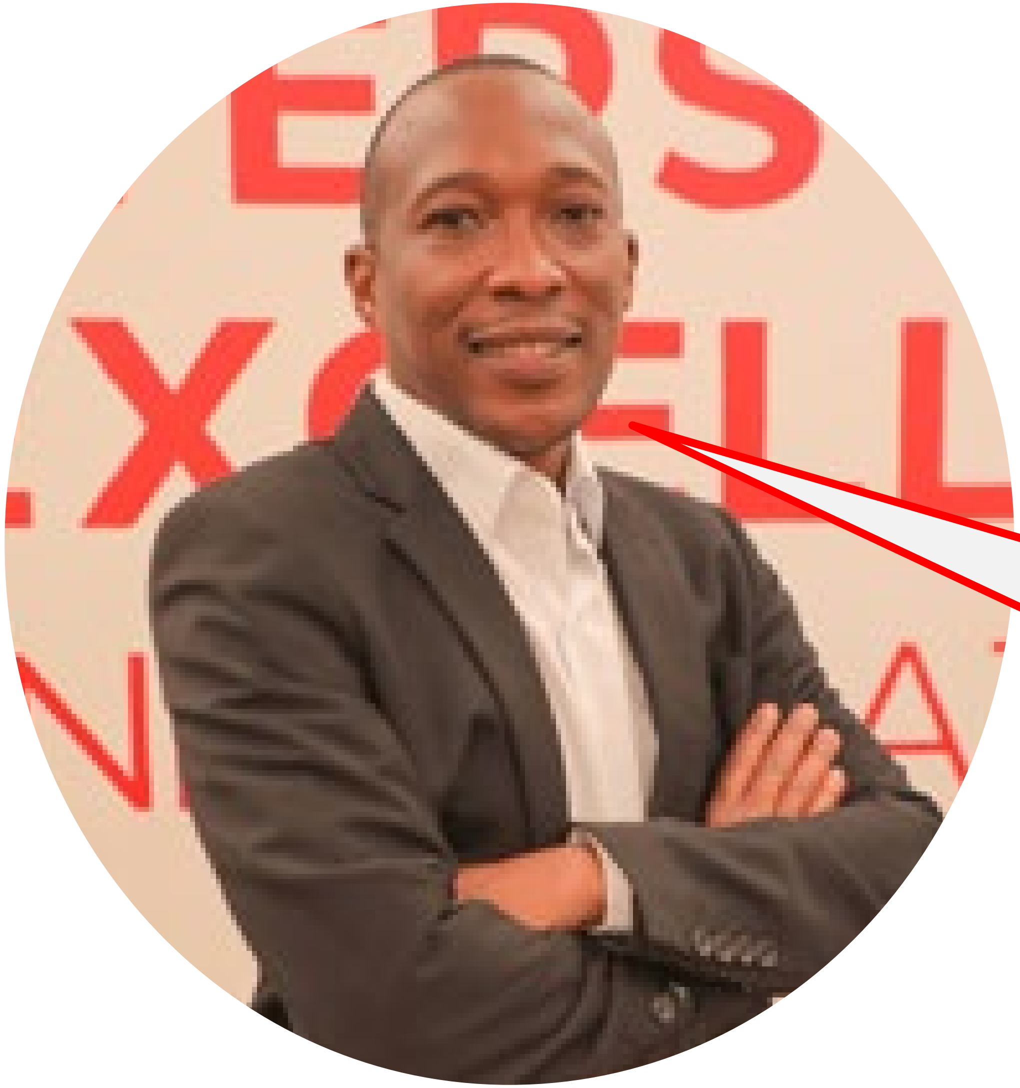

Thousands of Champions are needed to;

- Talk about the vaccine with their friends, family members, and neighbours
- Listen to the reservations and obstacles and help resolve them

Dr. Paul Buyego

“Let us encourage every one to vaccinate to prevent severe disease, hospitalization and death.”

Vaccination champions – what do they do?

1. **Ask about barriers** and do what you can to resolve them
2. **Share the facts** and clear the misinformation
3. **Offer to connect them** to vaccination centers
4. **Share that vaccines are safe** and easy to use
5. **Share your experience** about getting the vaccine

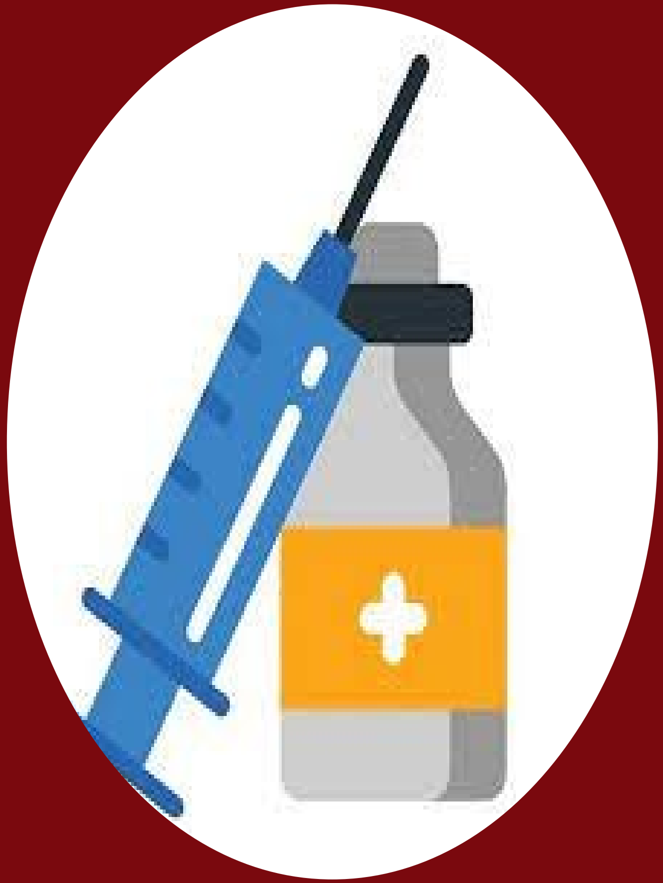

COVID-19

PART 1

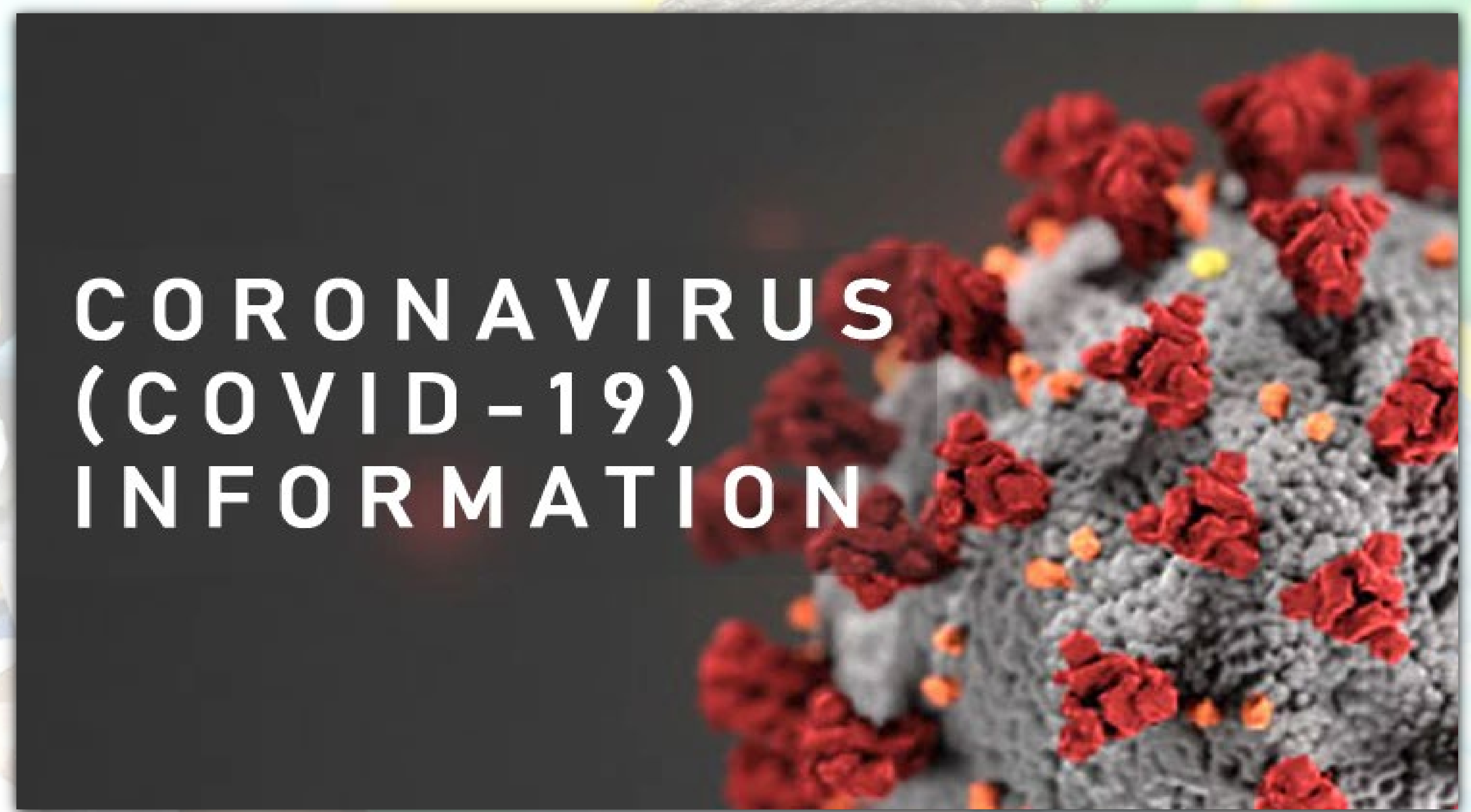

What is COVID-19?

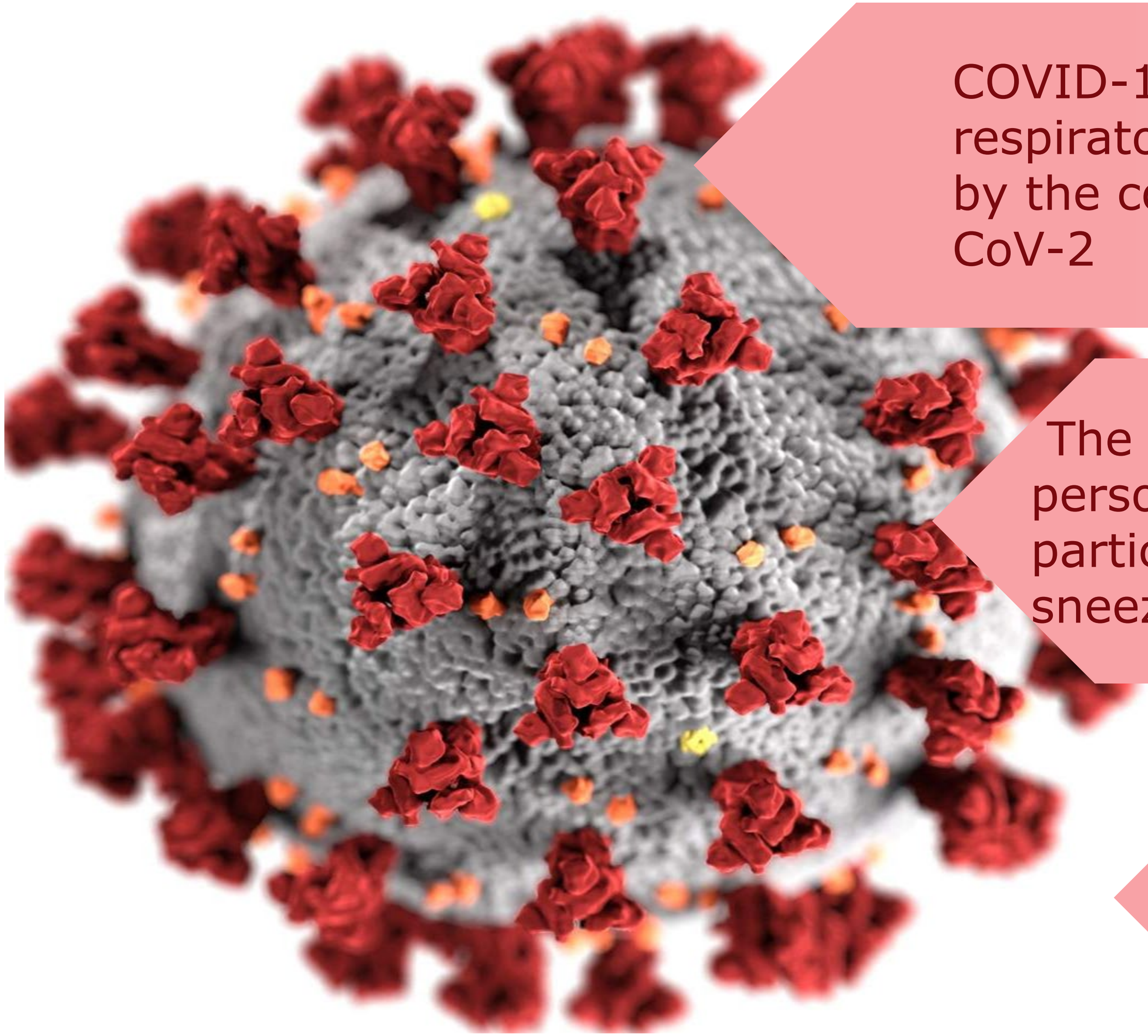

COVID-19 is an infectious respiratory disease caused by the coronavirus, SARS-CoV-2

The virus can spread from an infected person’s mouth or nose in small liquid particles (droplets) when they cough, sneeze, talk, sing or breathe heavily

Usually, the virus spreads when people are in direct or close contact (less than 1 meter) with an infected person

.0

What are signs and symptoms of COVID-19?

Common symptoms of COVID-19

- Mild fever,
- Cough,
- Fatigue/weakness/tiredness,
- Shortness of breath,
- Myalgias (muscle aches/pains).
- Complications/problems as a result of COVID-19 include severe disease and may lead to death

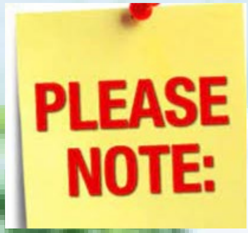

Anyone can become sick with COVID19, regardless of age and health status.

Older people above the age of 50 and those with underlying or people with chronic(long lasting) medical conditions are more likely to have severe forms of COVID-19

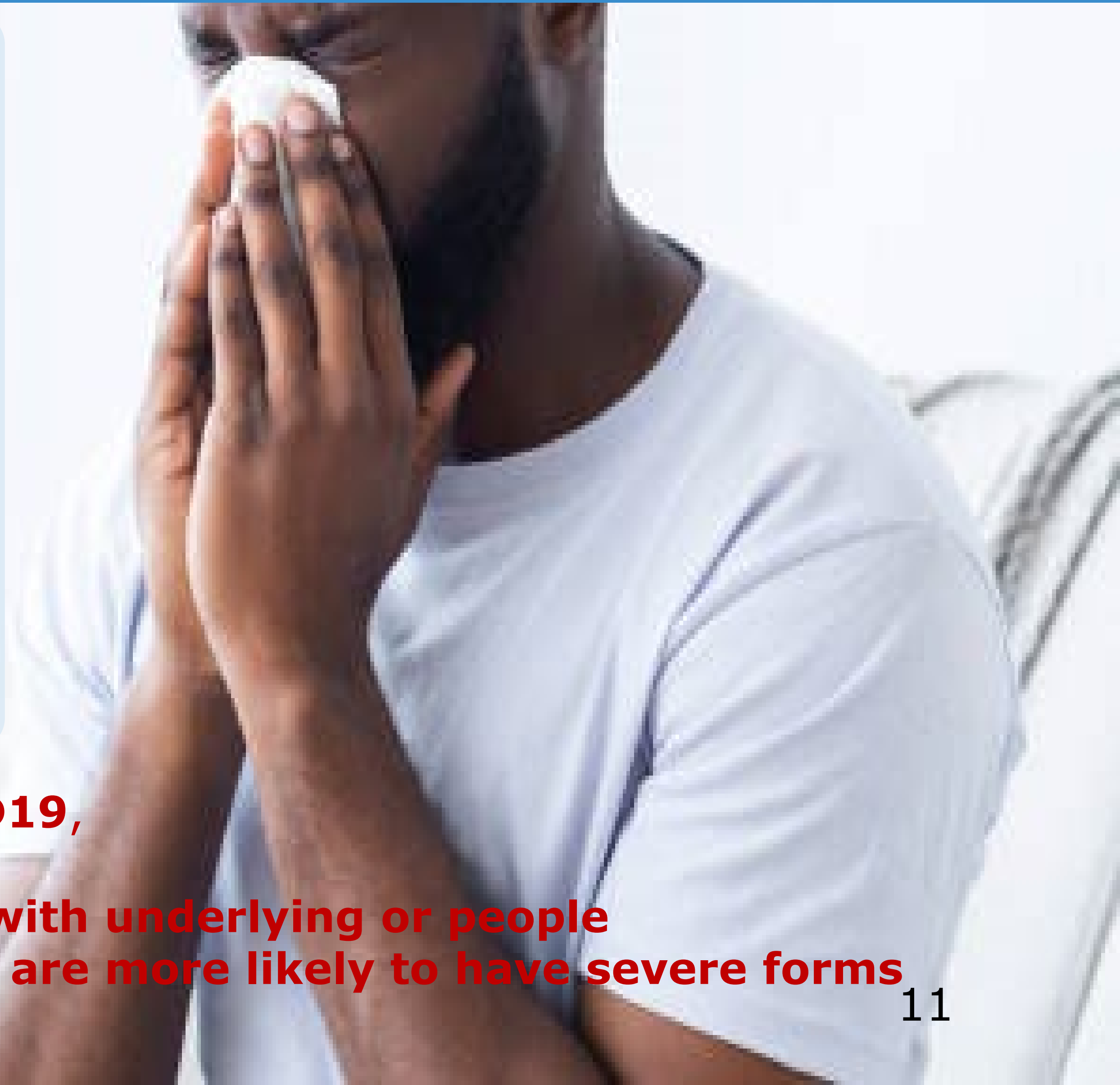

Priority populations for vaccination

Occupation

Are by the nature of their work exposed to COVID-19 infection

- Health workers,
- Security personnel
- Teachers and non-teaching staff in schools .

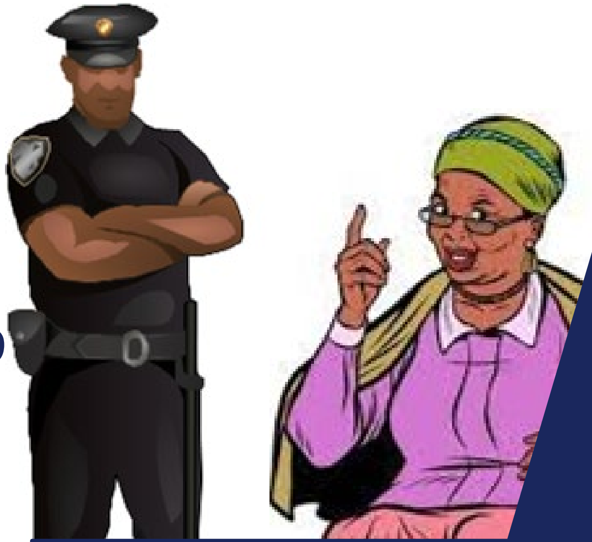

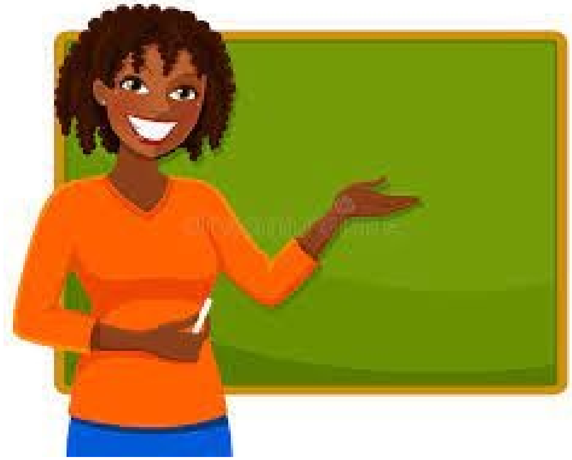

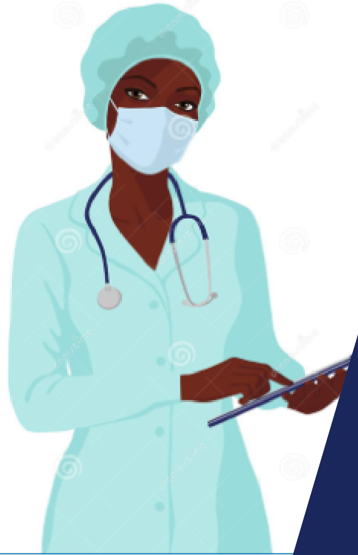

Weaker immune systems

People with weaker immune systems are more likely to need hospital care or die if they get COVID-19)

- Older people 50 years of age and above
- People with underlying conditions such as diabetes, hypertension, heart/liver/kidney disease, cancer and HIV.
- Adults from 18- 49 years of age and Children 12 -17 years with these underlying conditions are prioritized for vaccination

Special groups-Pregnant and Breast-feeding women

- The benefits of getting a COVID-19 vaccine far outweigh risks for individuals who are pregnant or might become pregnant in the future

- Vaccines are safe in breastfeeding mothers.

- The vaccines do not contain live virus, so being vaccinated does not pose a risk to the baby.

- If you are vaccinated for the coronavirus, there is no need to delay or discontinue breastfeeding

- The vaccines will not stop you from becoming pregnant in future. Women trying to conceive can be vaccinated with the current COVID-19 vaccines — there is no reason to delay pregnancy after completing the vaccine series.

Information to support patients and protect caregivers when managing COVID-19 at home

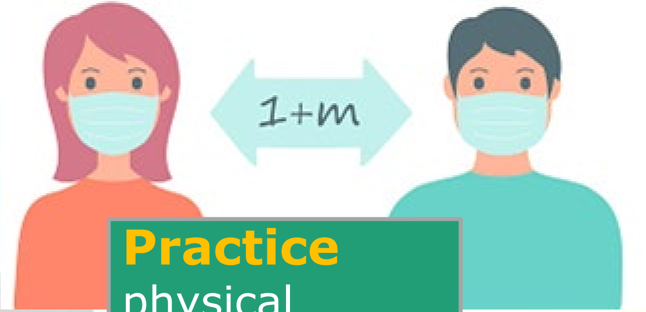

Practice physical distancing of at least 2 meters away from others

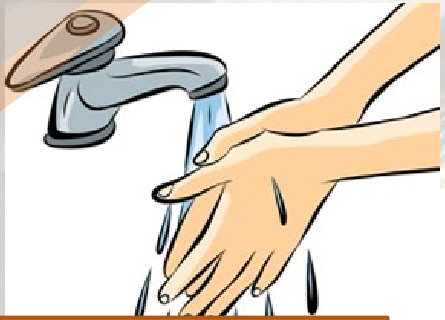

Wash your hands regularly with soap and clean water or use an alcohol-based hand sanitizer (at least 60% alcohol content)

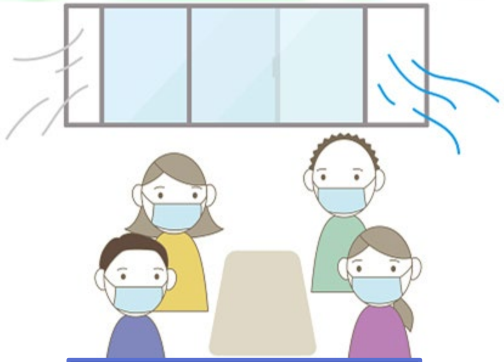

Keep rooms well ventilated with open windows

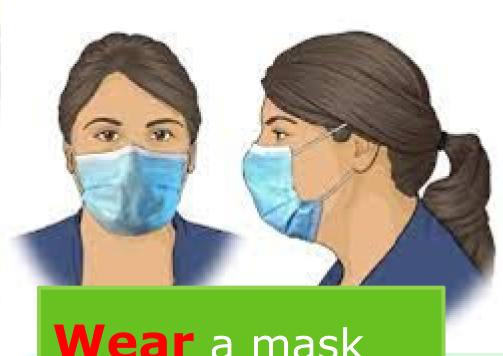

Wear a mask properly, covering your mouth and nose when in public, and while at home when you are caring for an infected person who is under home-based care/isolation

Information to support patients and protect caregivers when managing COVID-19 at home

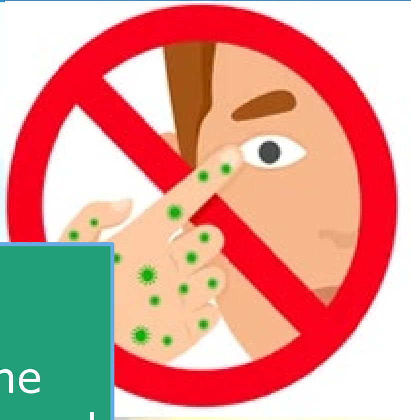

Avoid touching the eyes, nose and mouth

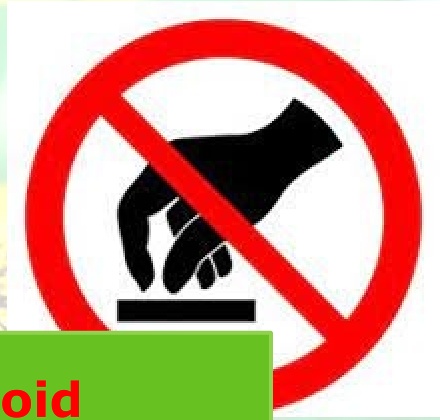

Avoid touching surfaces and clean surfaces regularly with standard disinfectants

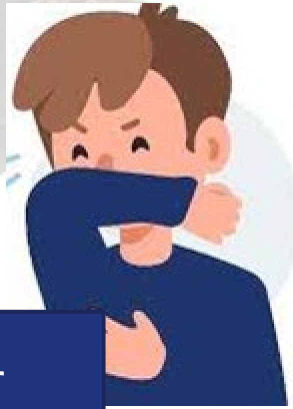

Cough or **sneeze** into a bent elbow or use a tissue if accessible.

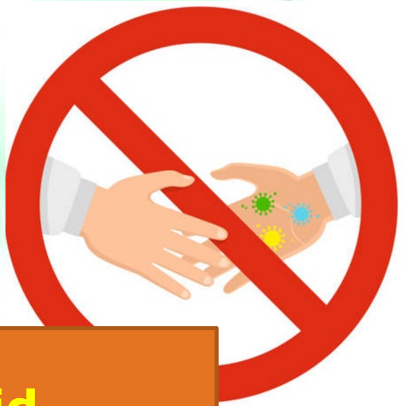

Avoid crowds, hand shaking and hugs

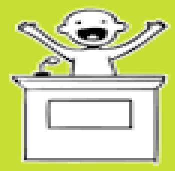

THE TAKE-HOME MESSAGE

- COVID-19 is an infectious/ contagious respiratory illness
- You can become infected by breathing in droplets from an infected person
- Common symptoms include; fever, shortness of breath these can worsen to more severe COVID-19 disease
- Anyone can get COVID-19, but older people >=50 years and those with underlying medical conditions are more likely to get severe forms of COVID-19 disease
- Most cases of COVID-19 are asymptomatic
- Observe all the SOPs for prevention of COVID-19

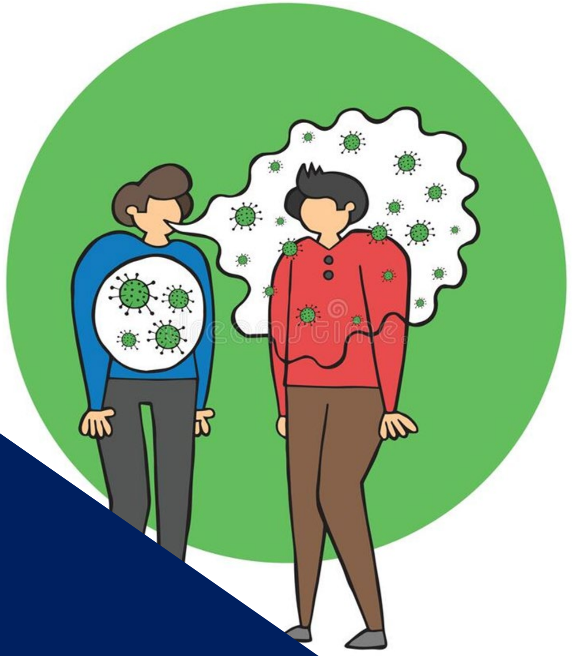

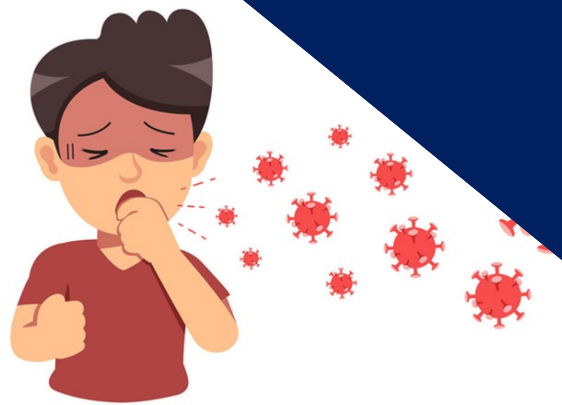

COVID-19 vaccines and vaccine safety

PART 2

Vaccines

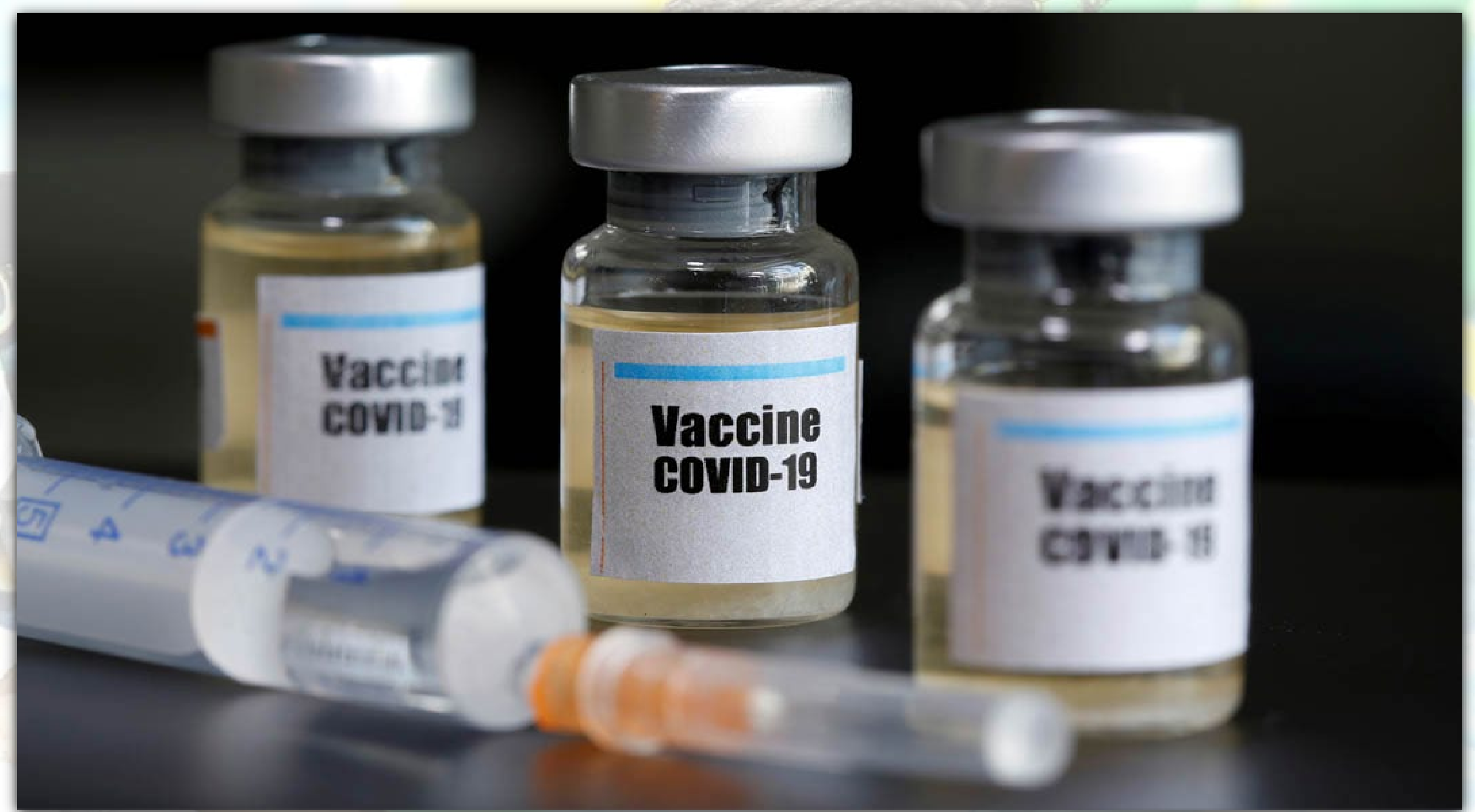

What is the goal of COVID-19 vaccination?

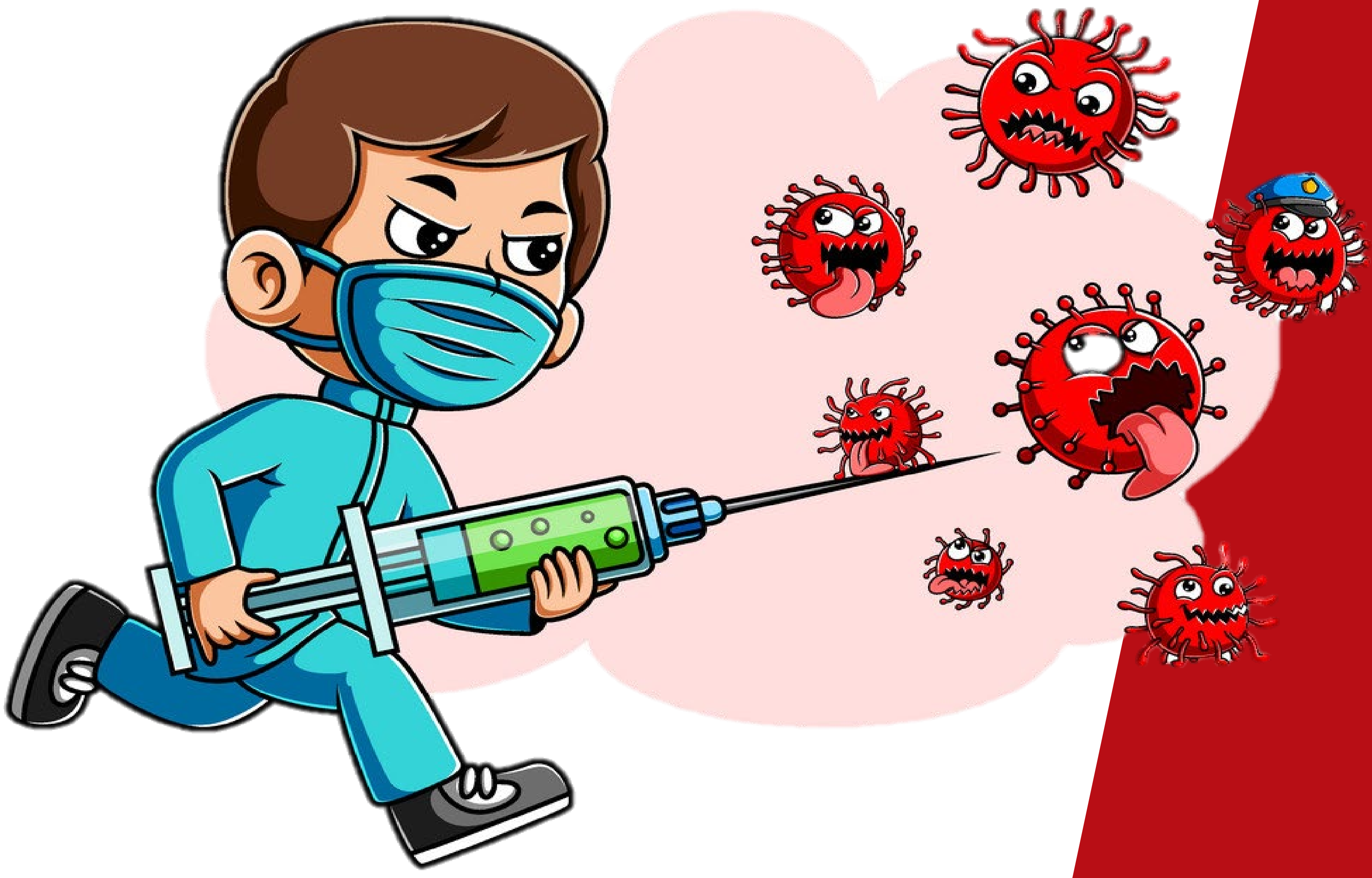

- To prevent and reduce **severe COVID-19 disease and deaths**
- Sustain national health system response
- Restore health and productivity of the Ugandan societies and the economy

Vaccines

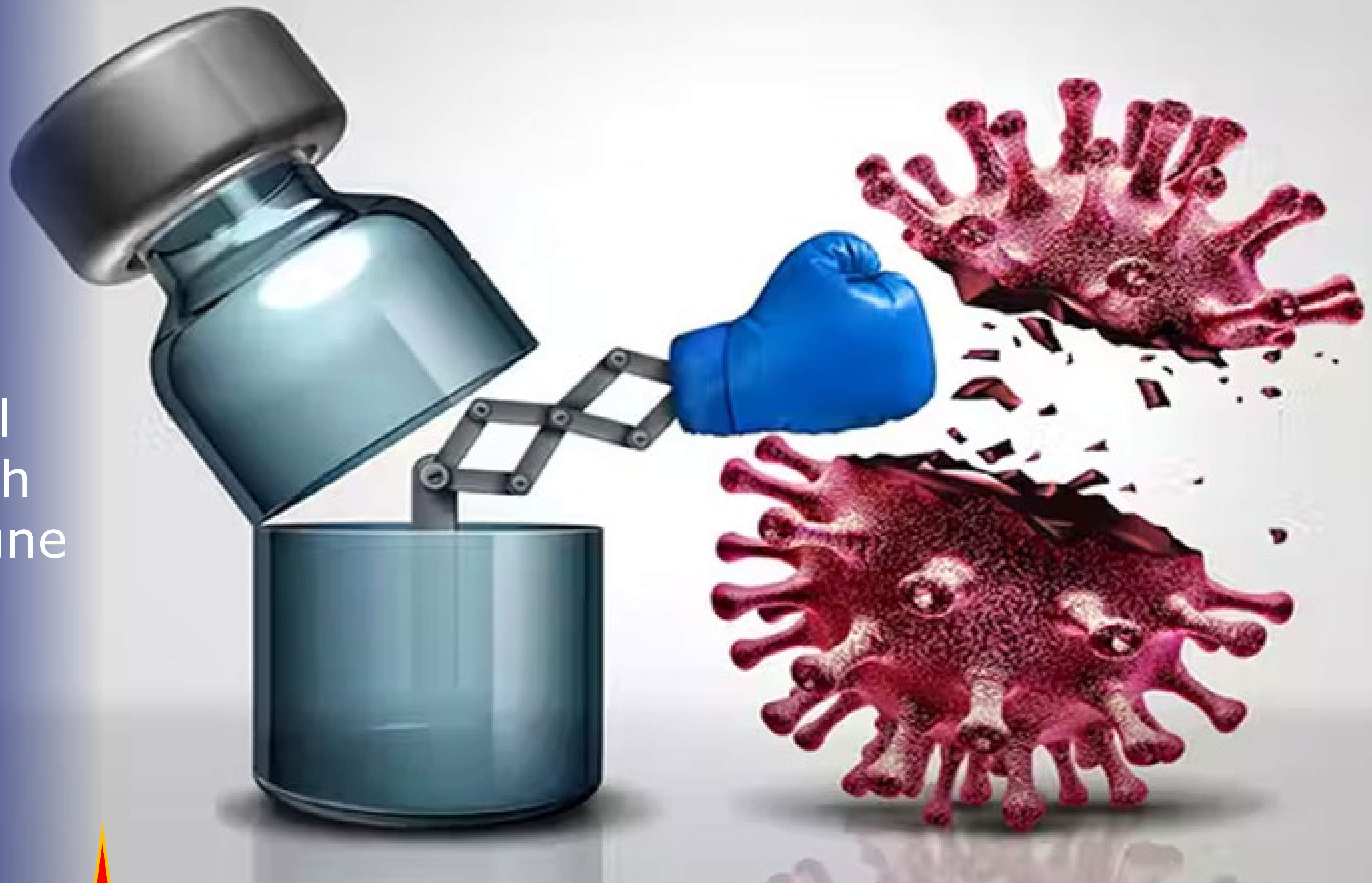

The vaccines for COVID-19 are all designed to teach the body's immune system to safely recognize and block the virus that causes COVID-19.

All vaccines have a very high efficacy/usefulness in preventing hospitalization, death and severe disease.

Types of Vaccines

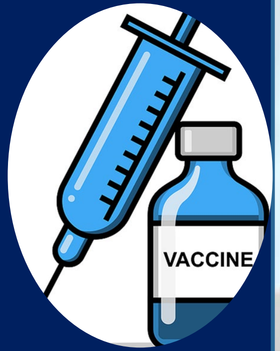

Inactivated or weakened virus vaccines: They use a weakened form of the virus that does not cause disease, but still generates an immune response. **(Sinovac, Sinopharm)**

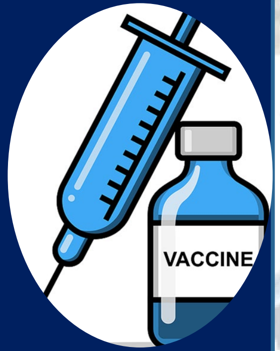

Viral vector vaccines: These use a virus that has been genetically engineered so that it can't cause disease but produces coronavirus proteins to safely prompt an immune response. **(AstraZeneca , Janssen)**

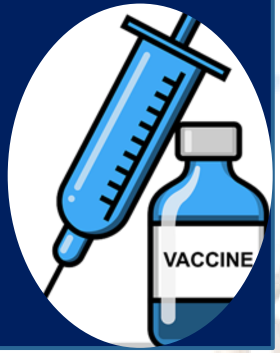

RNA and DNA vaccines: It is a new approach that uses genetically engineered RNA or DNA to generate a protein that safely prompts an immune response. **(Pfizer, Moderna)**

https://www.sfdph.org/dph/files/ig/vaccine/vaccine-ambassador-training-pdf.pdf

Pfizer & Moderna Vaccines: How They Work

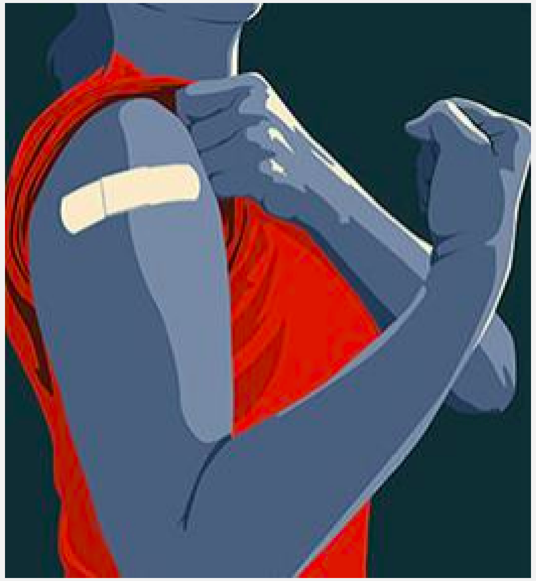

How a messenger RNA (mRNA) vaccine works. Scientists take some inactive virus genetic code that tells cells what to build and coat it in a lipid (fat) so it can enter the body's cells.

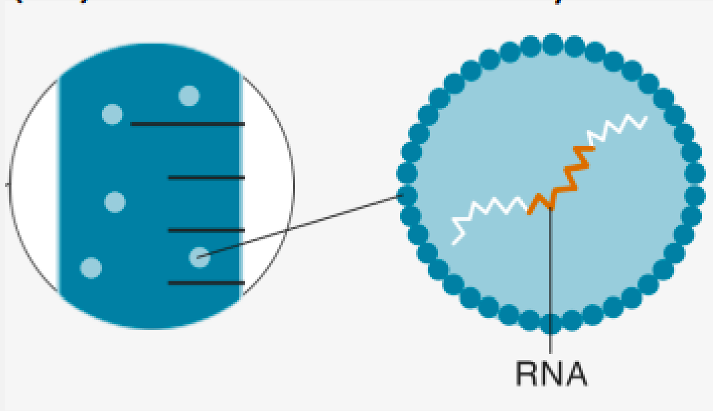

1

The vaccine enters the cells and tells the cell to produce the protein from the outside of the coronavirus so your body can recognize it later

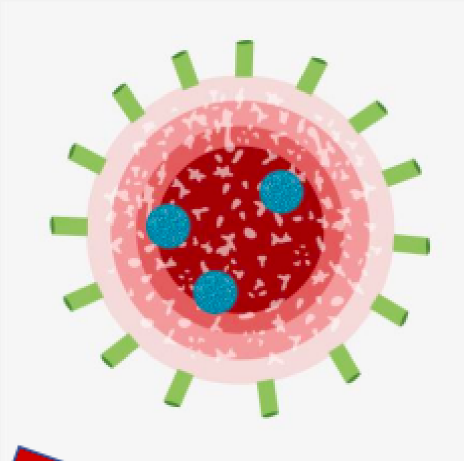

2

The immune system produces antibodies that recognize the protein from the outside of a coronavirus

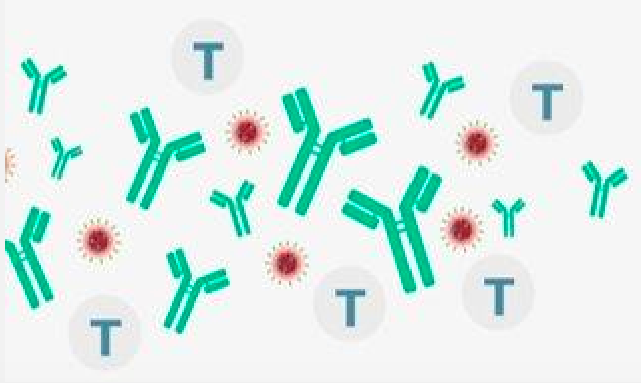

3

If your body comes in contact with a coronavirus the immune system can now recognize it and fight the virus.

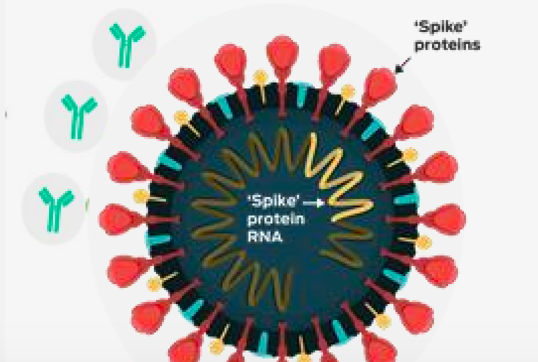

COVID-19 vaccines available in Uganda

Sinovac

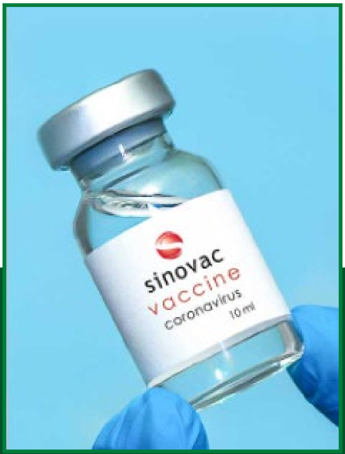

Pfizer

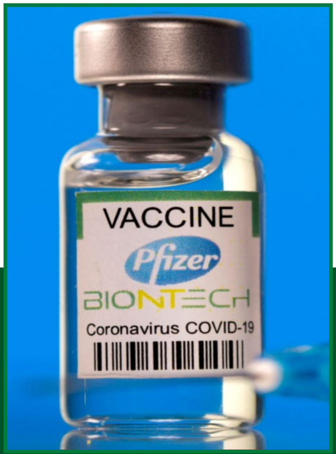

J&J

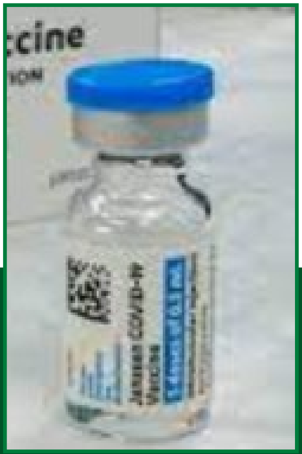

Moderna

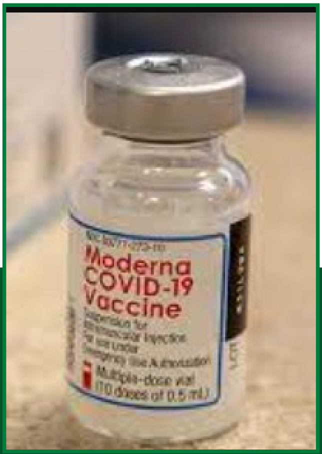

Sinopharm

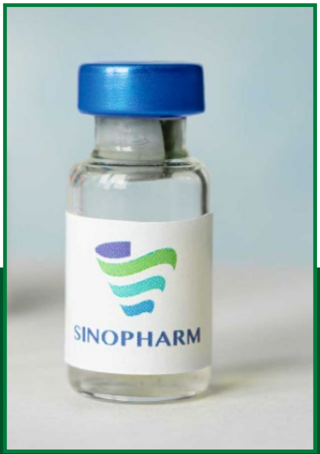

AstraZeneca

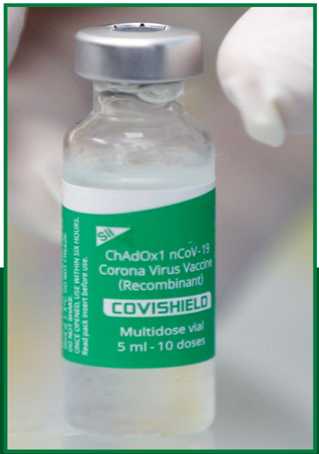

COVID-19 Vaccination Models in place for better Targeting of priority population

Point of Care

Leveraging on routine care facilities to target mobilization and vaccination i.e. PLHIV and PWC at NCD clinics

Place of Worship

Religious leaders mobilizing the elderly and PWC through religious leaders for vaccination at places of worship as designated outreach vaccination centres

Vaccination Champions Model

Developing content to training selected KoL n appropriate strategies for community-led mobilization for vaccination

Place of Work Model

Vaccinate a working to reach an older person model; Using workplaces to mobilize familes for vaccinations

All COVID-19 vaccines can be administered to:

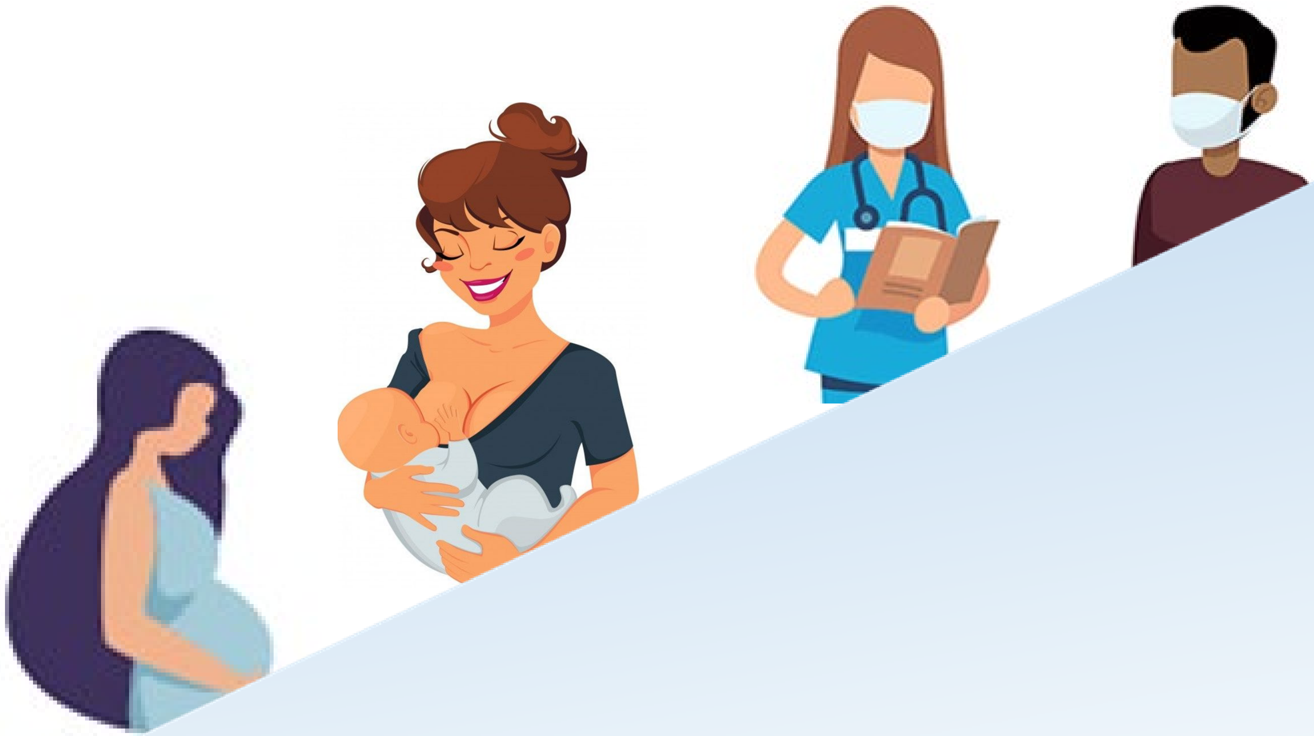

- **People aged 18 years and above.**
- **Pregnant and lactating women**
- **People with long lasting and underlying illness (from age 12 and above)**
- **People that have recovered from COVID 19.**
  - Given after 3 months
  - No need for COVID-19 testing

Matching, mixing and booster vaccinations (MoH guidance)

Vaccine scheduling— where the 2nd dose is different from the 1st dose

Alternative vaccine boosting –vaccine used for a booster dose may differ from the vaccines used in the initial full vaccination or even for vaccines used under the primary schedule as above.

.

Boosting recommended after 6 months for fully vaccinated individuals in the following categories

- All adults above 50 years
- All HCW, teachers and non-teaching , security personnel
- People with co-morbidities.
- All fully vaccinated people.

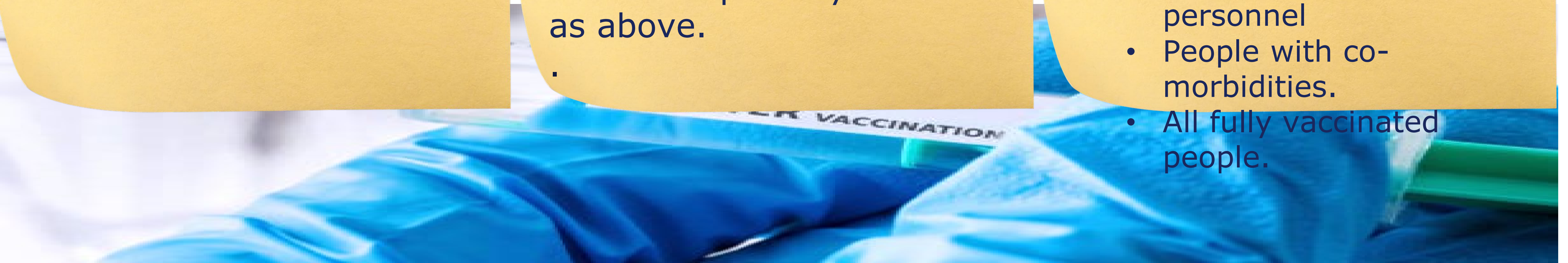

VACCINE ADMINISTRATION						
**Vaccine Name**	**Dosage**	**Dose**	**Dose interval**	**Device**	**Route of Admin.**	**Site of Admin.**
Astrazeneca	0.5 ml	2 doses	8 - 12 weeks	0.5 ml auto-disable (AD) syringe	**Intramuscular (IM)**	**Left Deltoid Muscle**
Sinovac	0.5 ml	2 doses	4 weeks	0.5 ml auto-disable (AD) syringe		
Pfizer-BioNTech	0.3 ml	2 doses	3 - 4 weeks	0.3 ml auto-disable (AD) syringe		
Moderna	0.5 ml	2 doses	4 weeks	0.5 ml auto-disable (AD) syringe		
Janssen	0.5 ml	1 dose	N/A	0.5 ml auto-disable (AD) syringe		
Sinopharm	0.5 ml	2 doses	3 - 4 weeks	0.5 ml auto-disable (AD) syringe		

The dose interval is 12 weeks for AZ and 4 weeks for all the rest, except J&J which is only one dose

Eligible categories for COVID 19 booster dose

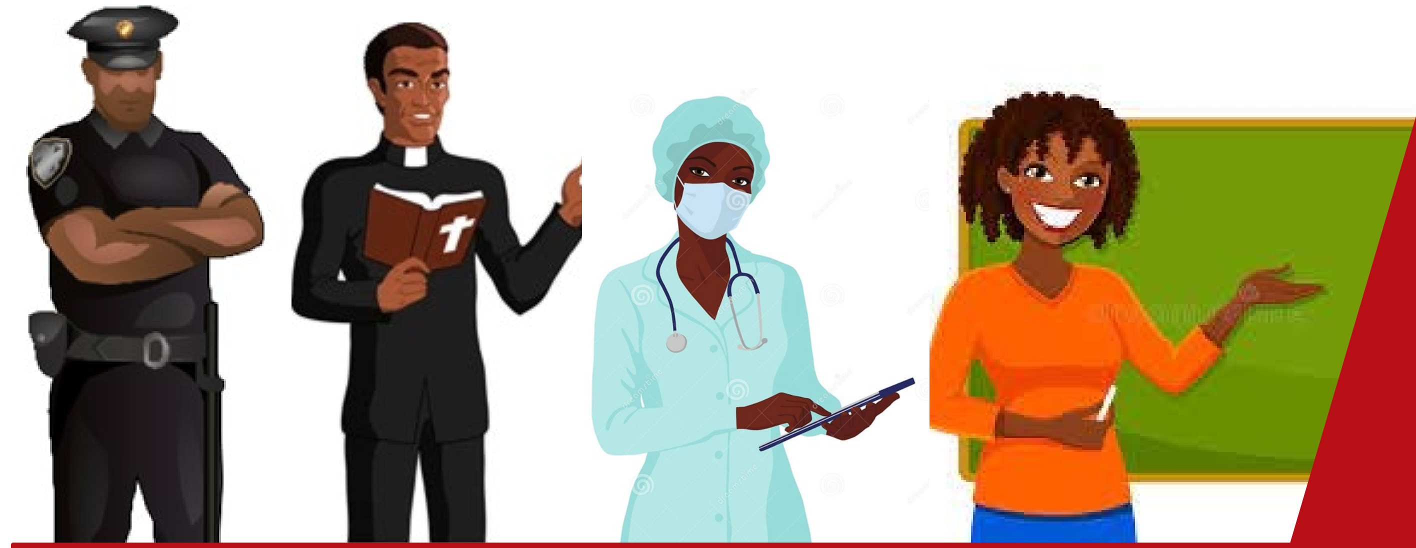

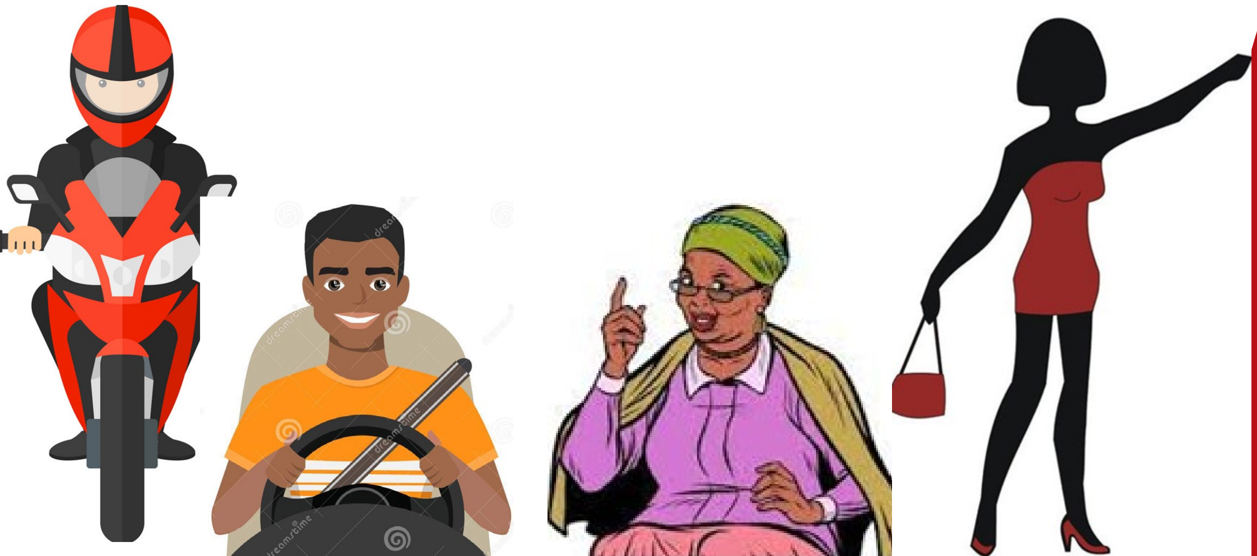

- All those aged 50 years and above
- Health workers
- Teachers both in pre-primary, primary, secondary and tertiary institutions
- Religious leaders
- Cultural leaders
- Security personnel
- Media
- Drivers and conductors of public transport vehicles
- Bodaboda riders
- Bar and night club workers
- Market workers and vendors

Completion of Primary Series of COVID-19 Vaccines (Matching and mixing/Booster dosing)

	First Dose	Second Dose	Interval between doses
1	AstraZeneca	Pfizer or Moderna	≥8 weeks following dose 1 of AZ
2	Pfizer	AstraZeneca	≥ 4 weeks following dose 1 of Pfizer
3	Moderna	AstraZeneca	≥ 4 weeks following dose 1 of Moderna
4	Sinopharm	AstraZeneca or Pfizer or Moderna	≥ 4 weeks following dose 1 of Sinopharm
5	Sinovac	AstraZeneca or Pfizer or Moderna	≥ 4 weeks following dose 1 of Sinovac

How do we know if COVID-19 vaccines are safe?

COVID-19 vaccines go through a **rigorous, multi-stage** and **testing** before approval.

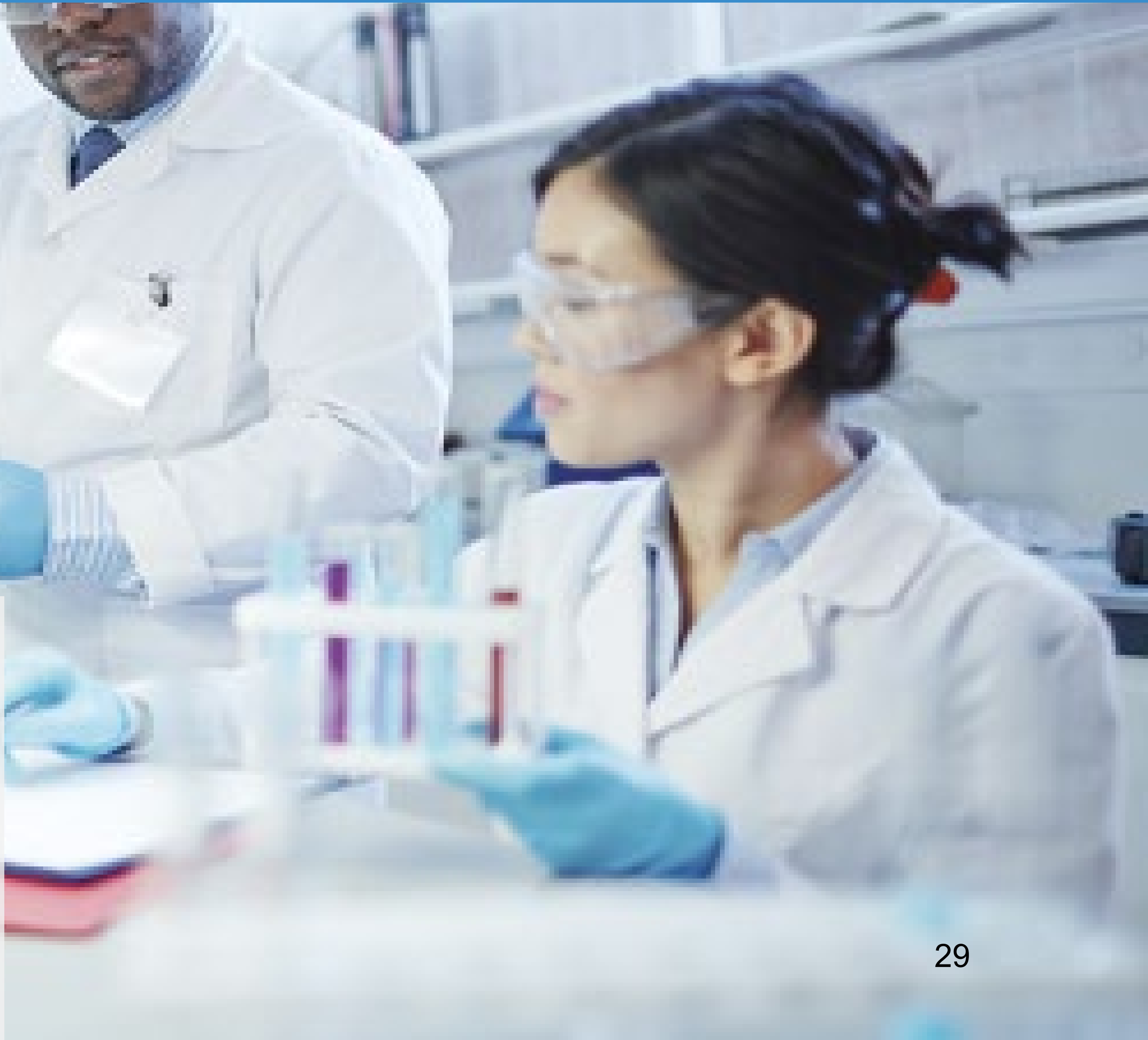

The National Drug Authority is responsible for the safety and quality of medicines in Uganda and **has approved the vaccines** for use **after a rigorous process of evaluation and approval by the World Health Organization and MOH Uganda.**

Most side-effects are mild and resolve in 1- 2 days

Common side effects:

- Fever
- Headache
- Pain at injection site
- Nausea
- Vomiting
- Decreased appetite
- Fatigue
- Tiredness
- Dizziness
- Body chills

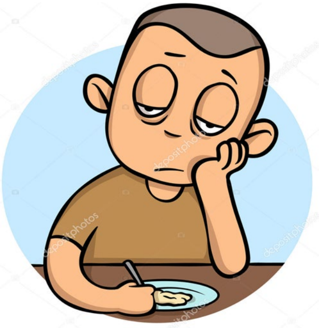

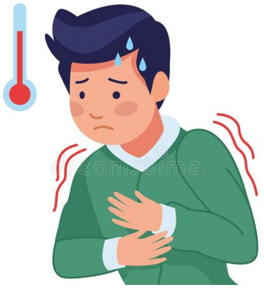

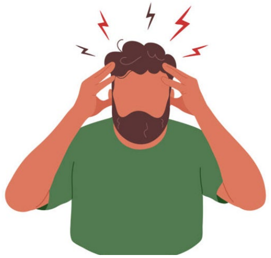

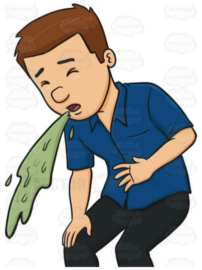

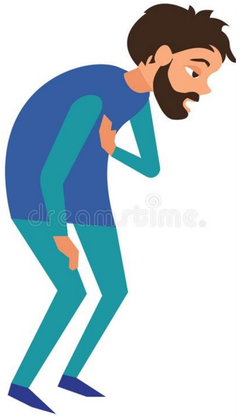

- Clotting disorder
- Heart problems
- Lymph node swelling

Rarely reported side effects:

Report any side-effect you may experience to NDA via

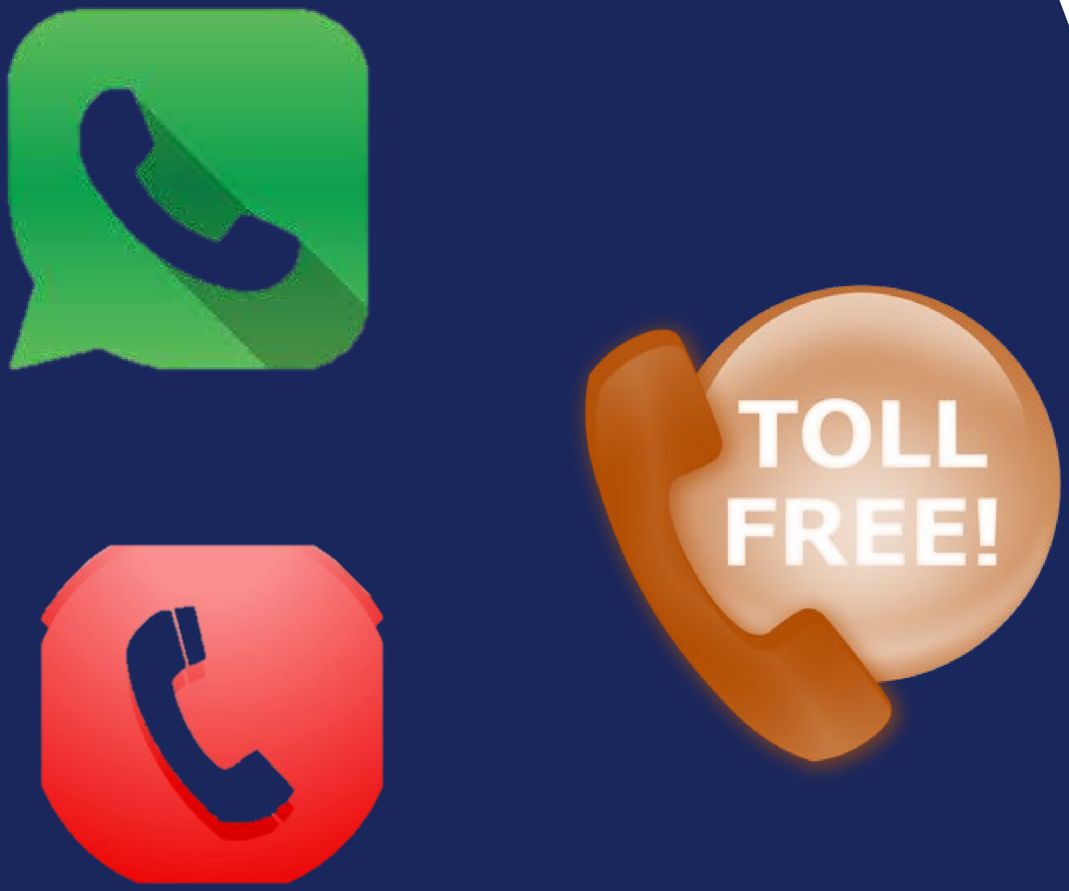

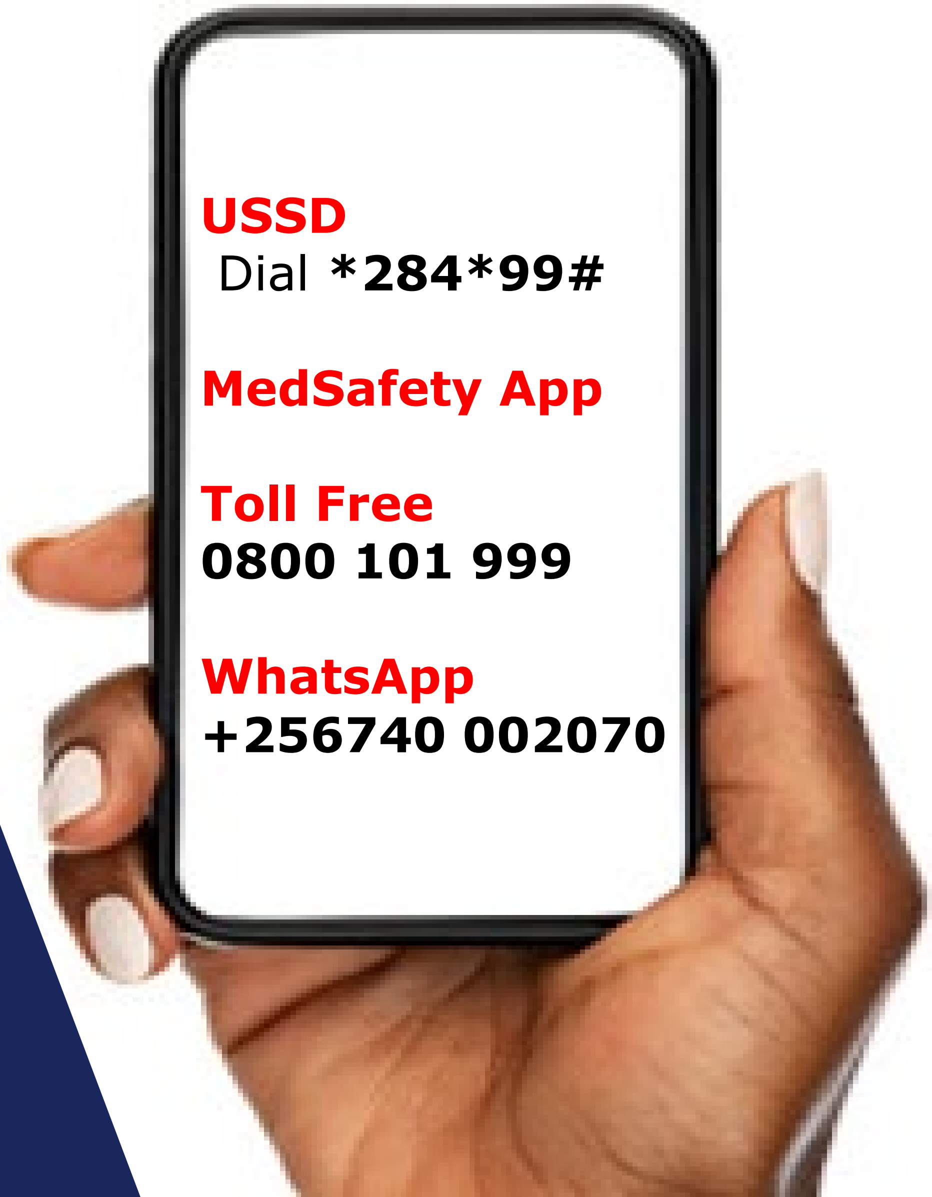

USSD
Dial ***284*99#**

MedSafety App

Toll Free
0800 101 999

WhatsApp
+256740 002070

Don’t forget to include the following in your report

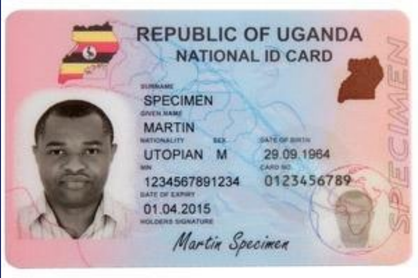

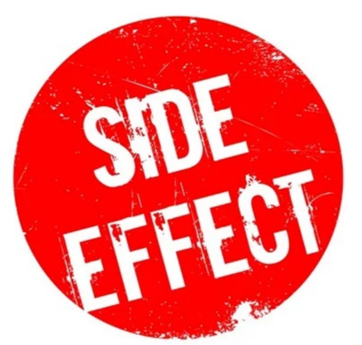

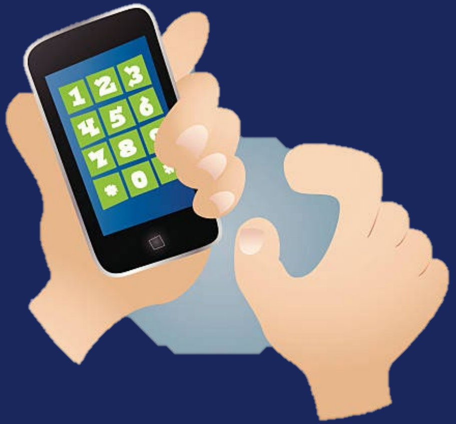

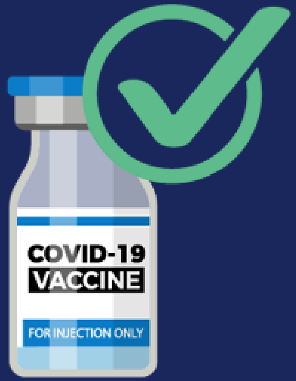

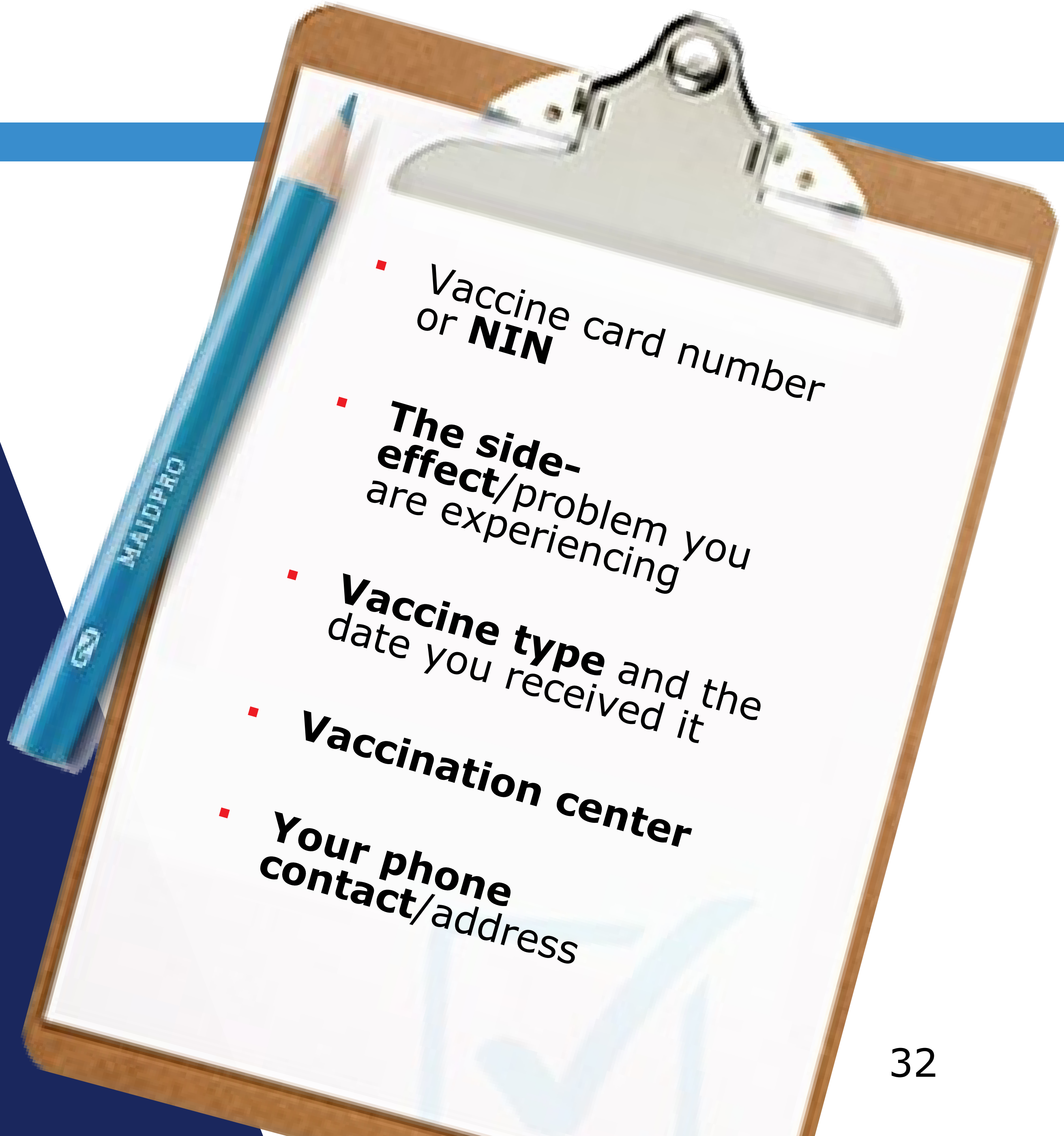

- Vaccine card number or **NIN**
- **The side-effect**/problem you are experiencing
- **Vaccine type** and the date you received it
- **Vaccination center**
- **Your phone contact**/address

Vaccine PV Interventions cont'd

Animation video; Vaccination safety and reporting

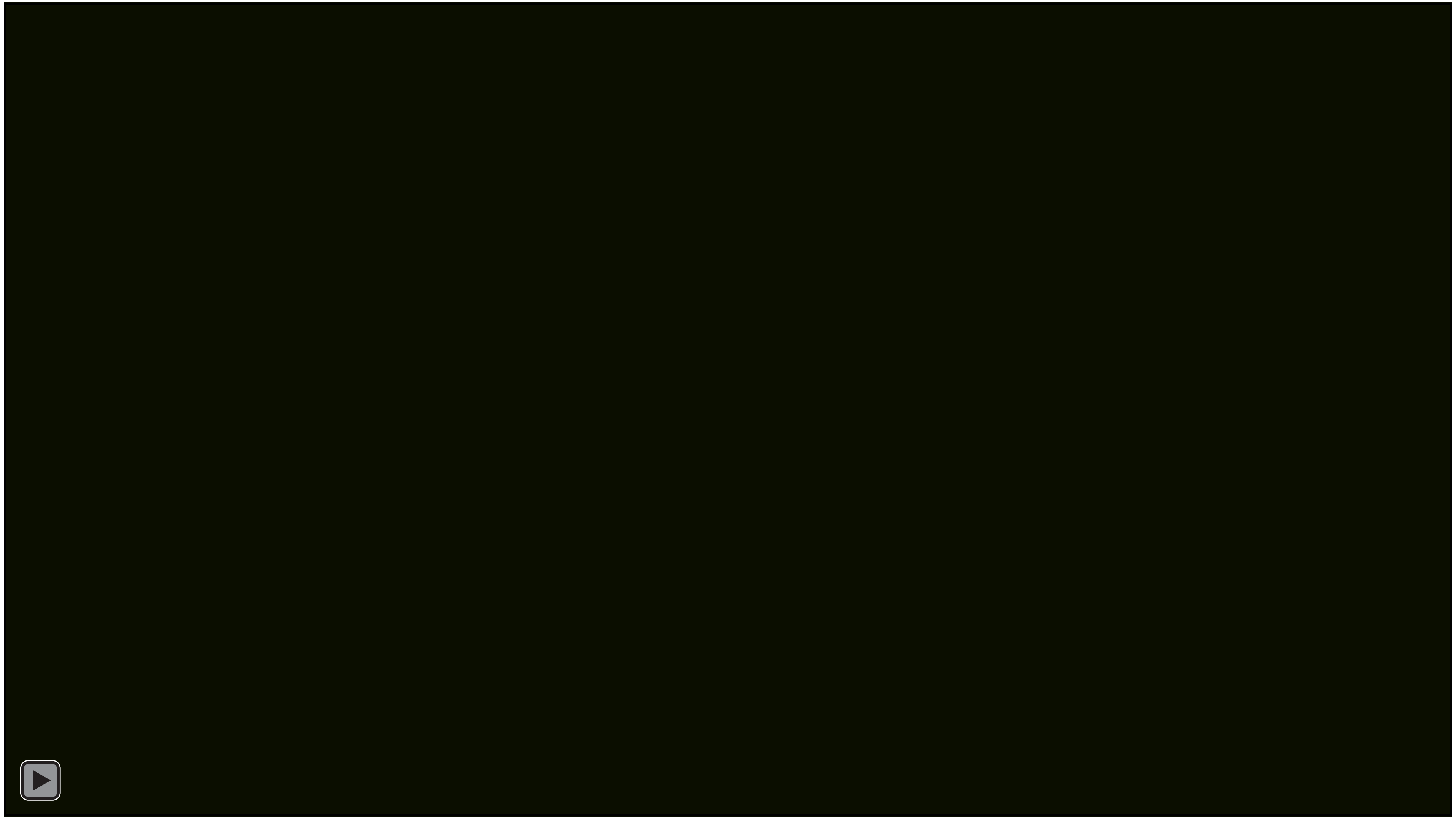

Continue to observe other SOPs

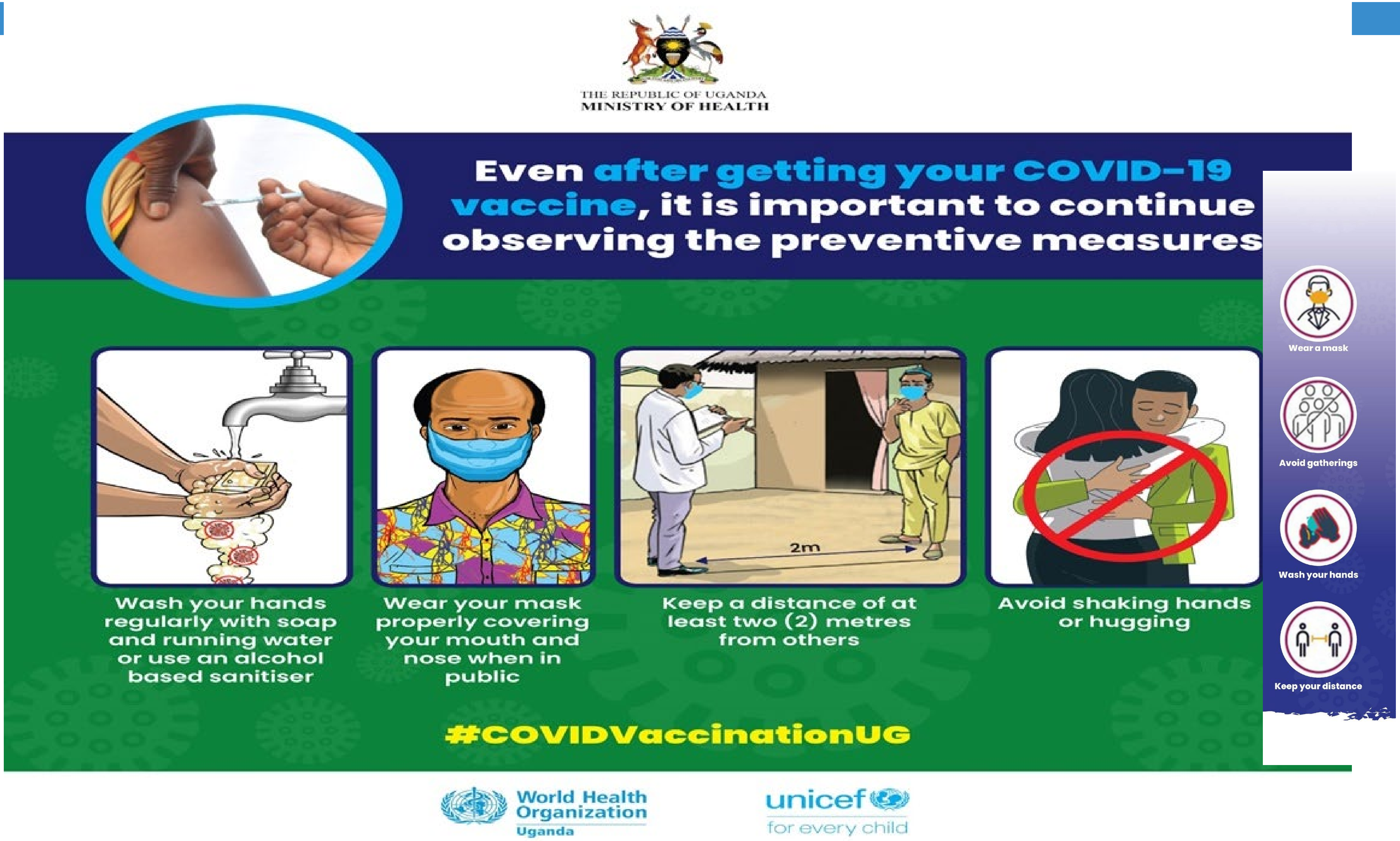

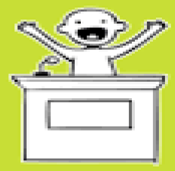

THE TAKE-HOME MESSAGE

- COVID-19 vaccines are safe, effective and free
- These vaccines have been approved for use in Uganda by the WHO and MoH
- All vaccines are highly effective against COVID-19

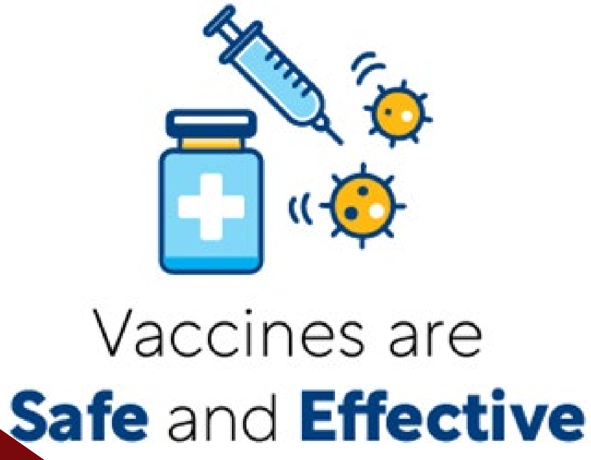

- COVID-19 vaccines are ONLY recommended for people aged 18 years and above.In Uganda Only Pfizer is recommended for children aged 12 years and above.
- Vaccines can be found in designated sites as are routinely communicated by MoH
- Report any side-effects/problems related to the vaccine to the NDA

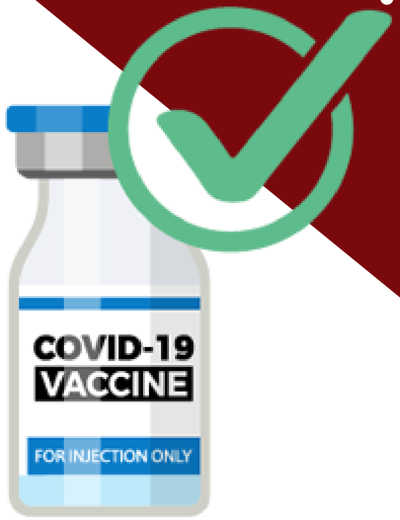

How to talk about vaccines

PART 3

Communication

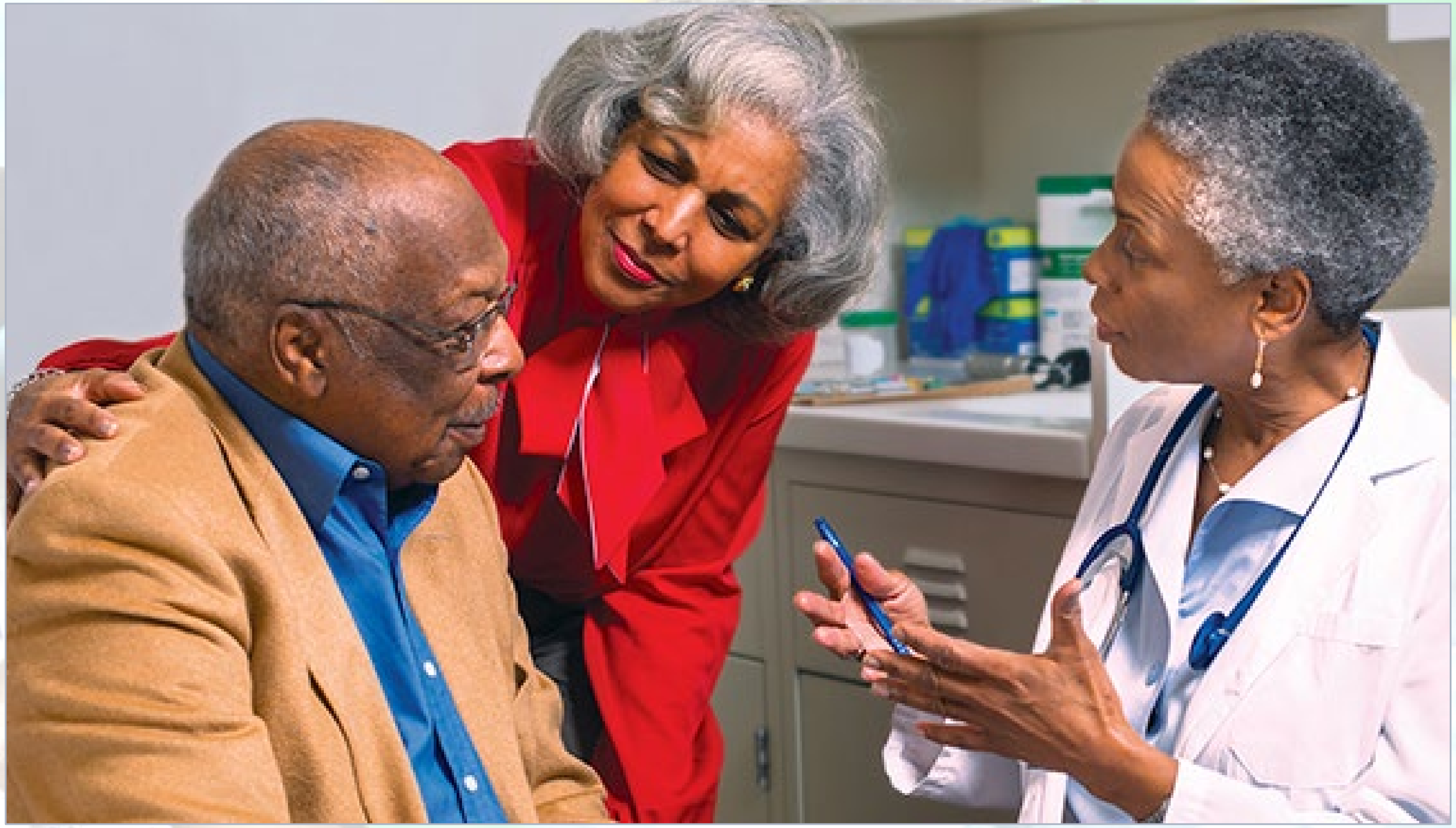

https://www.who.int/news-room/feature-stories/detail/how-to-talk-about-vaccines

1.

Listen with empathy

And acknowledge how they're feeling.

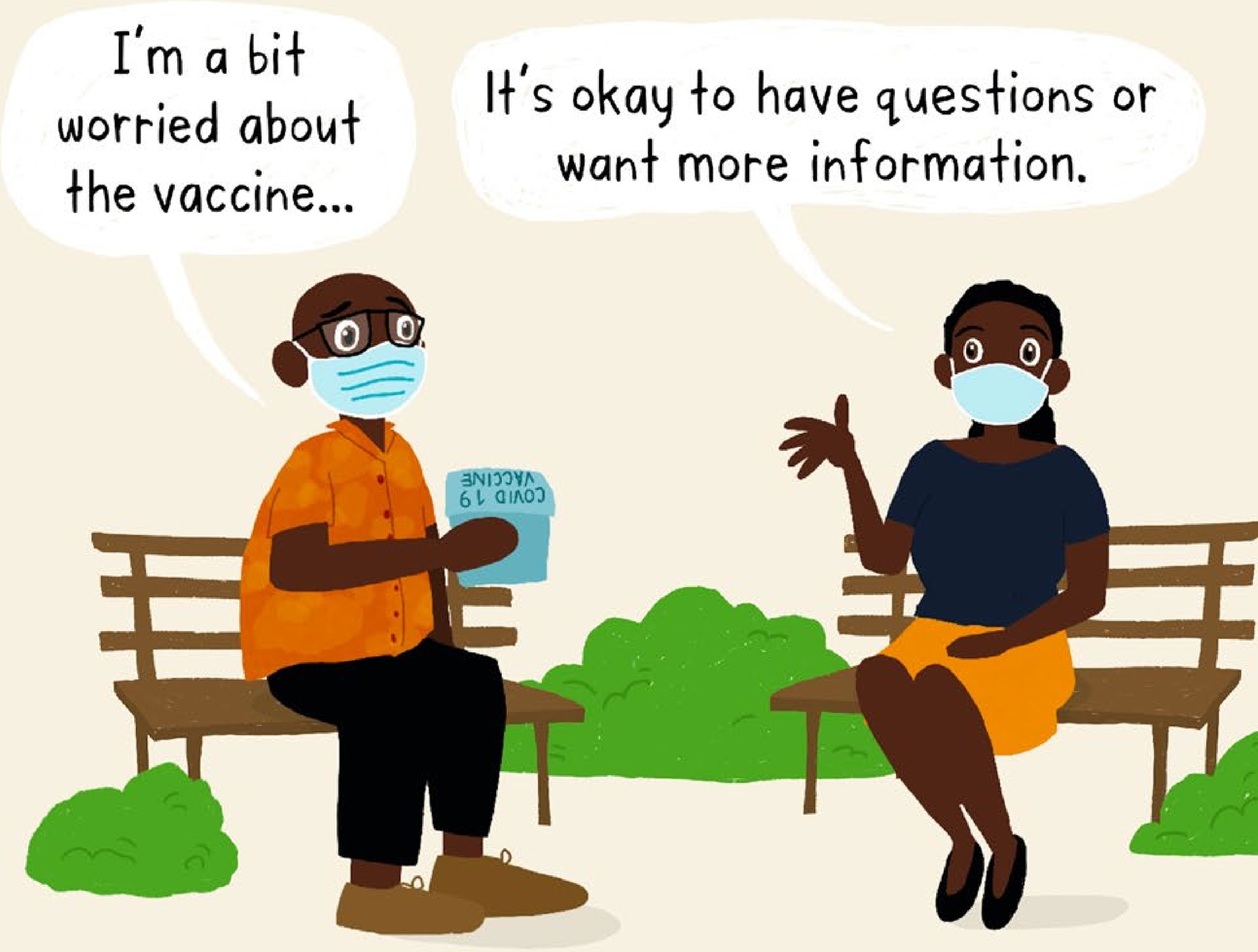

2.

Ask open-ended questions

To help you understand their concerns

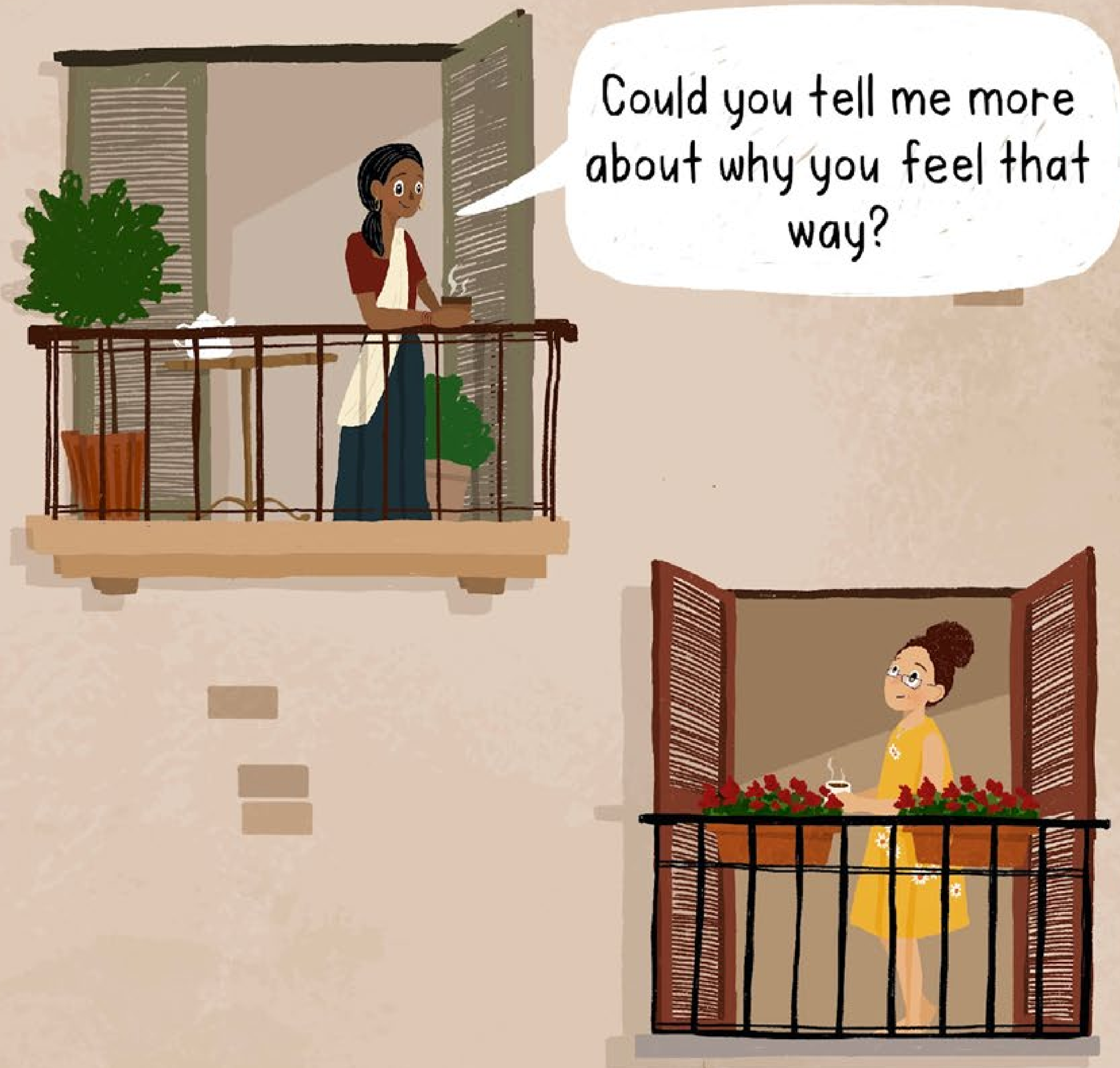

3.

Share trusted information

Visit the WHO website or chat to your doctor or nurse to find answers to common questions

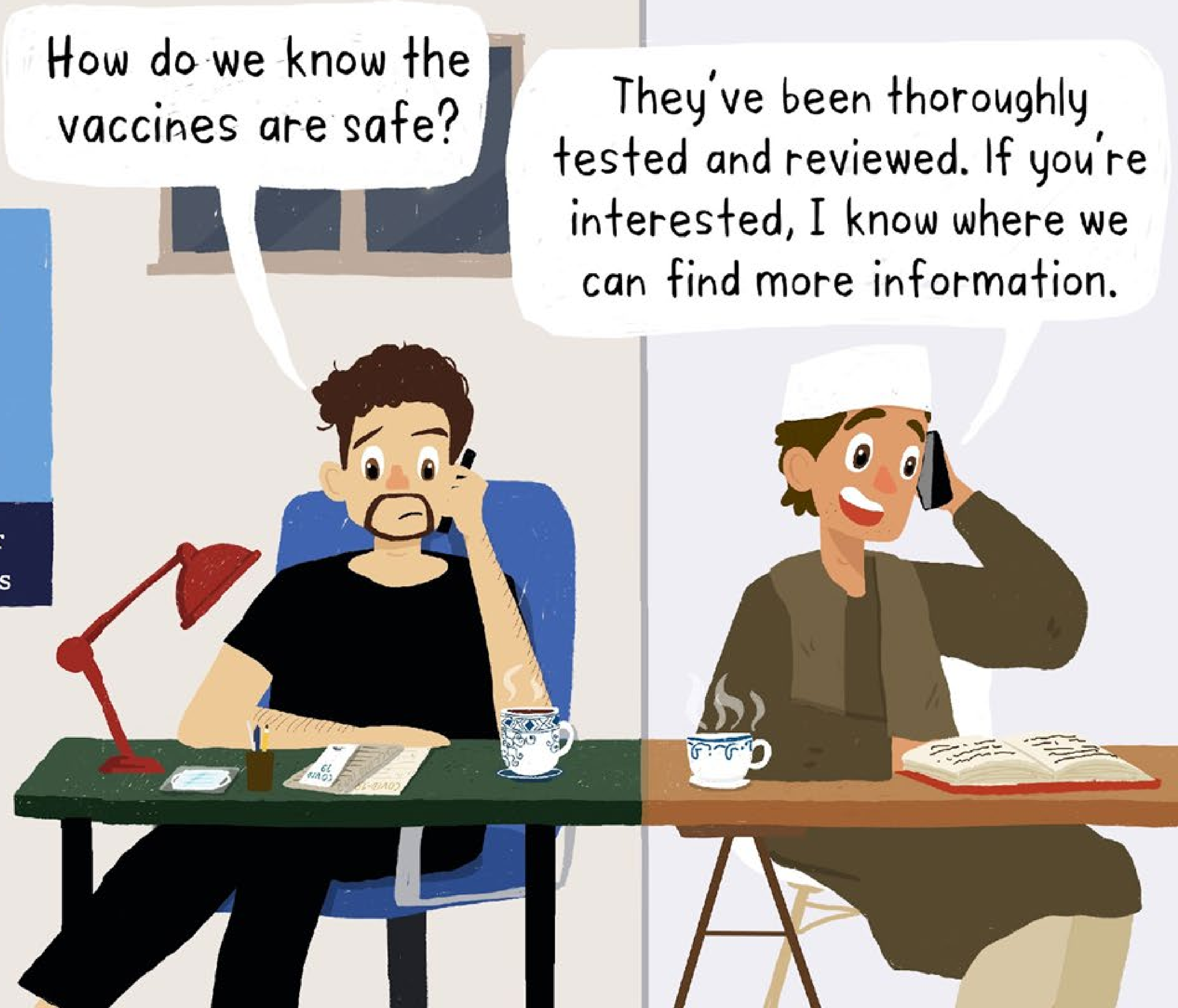

4.

Explore reasons for wanting to get vaccinated

Share your motivations and what helped you overcome any concerns.

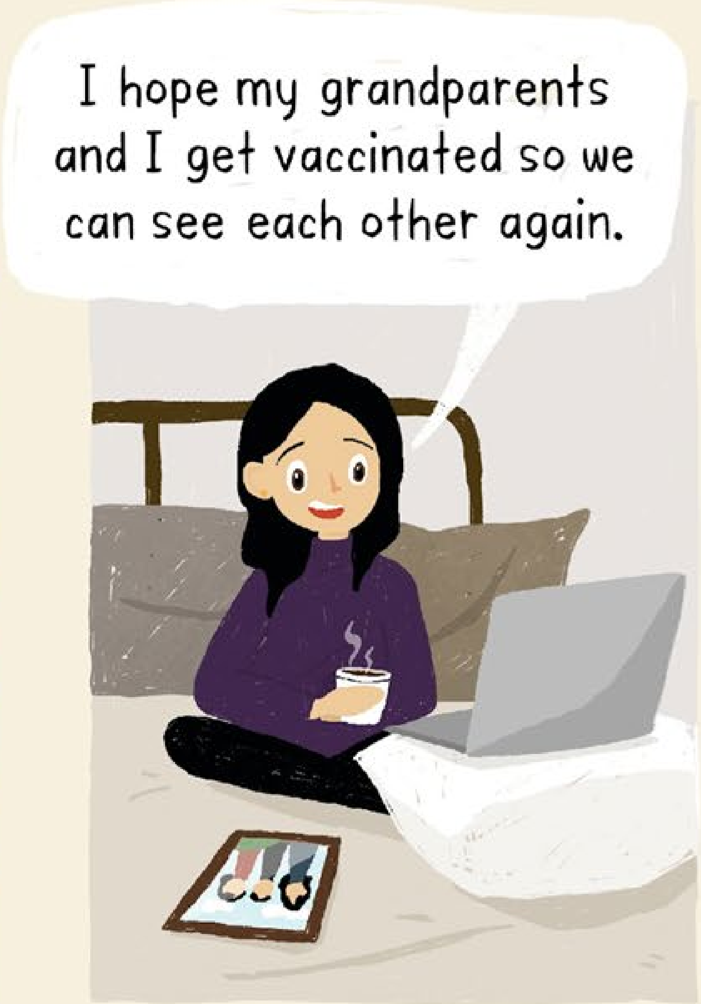

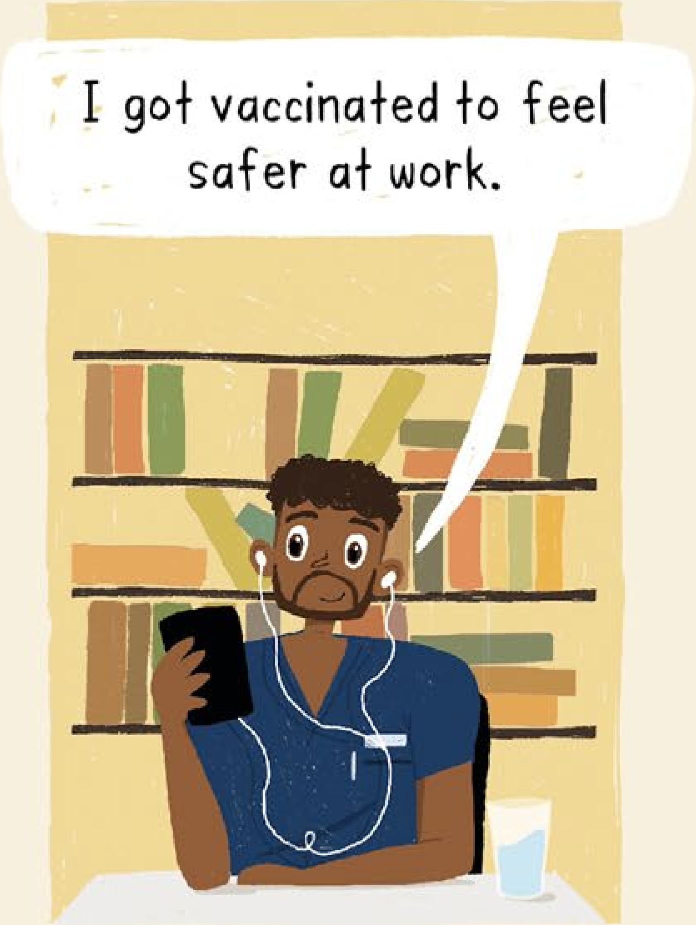

Strategies for vaccine communication (cont'd)

Tailor	You know your audience: tailor your message to them
Utilize	Utilize trusted sources – including testimonials of respected figures (I got my vaccine buttons, social media)
Provide	Provide clear info on vaccine access
Acknowledge	Acknowledge people's fears and concerns- especially related to trauma and racism in healthcare
Explain	Explain benefits of getting the vaccine, not just the consequences of not doing it

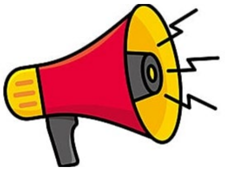

Strategies for vaccine communication

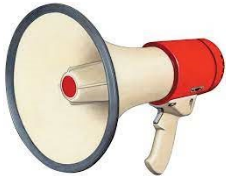

Recognize	Recognize people may need to hear the messages multiple times. Avoid amplifying/repeating misinformation
Avoid	Avoid judgment, embrace curiosity to understand
Avoid	Avoid shaming
Don't talk about	Don't talk about pharmaceutical companies, talk about people behind the vaccines: scientists & doctors

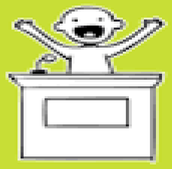

THE TAKE-HOME MESSAGE

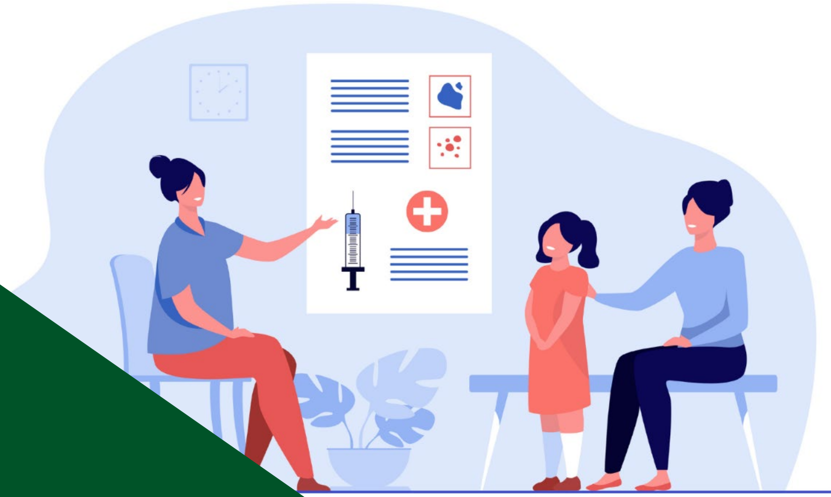

- Listen with empathy
- Ask open ended questions
- Share or refer to trusted sources of information
- Explore reasons for wanting to get vaccinated

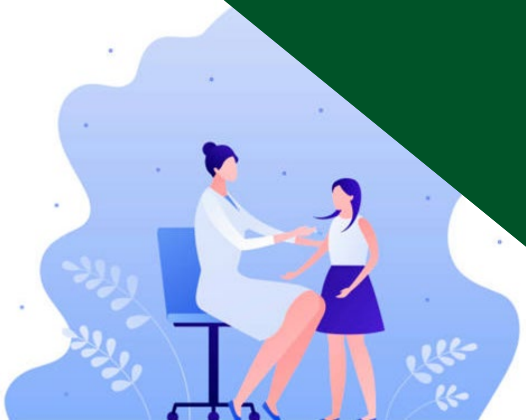

Sources

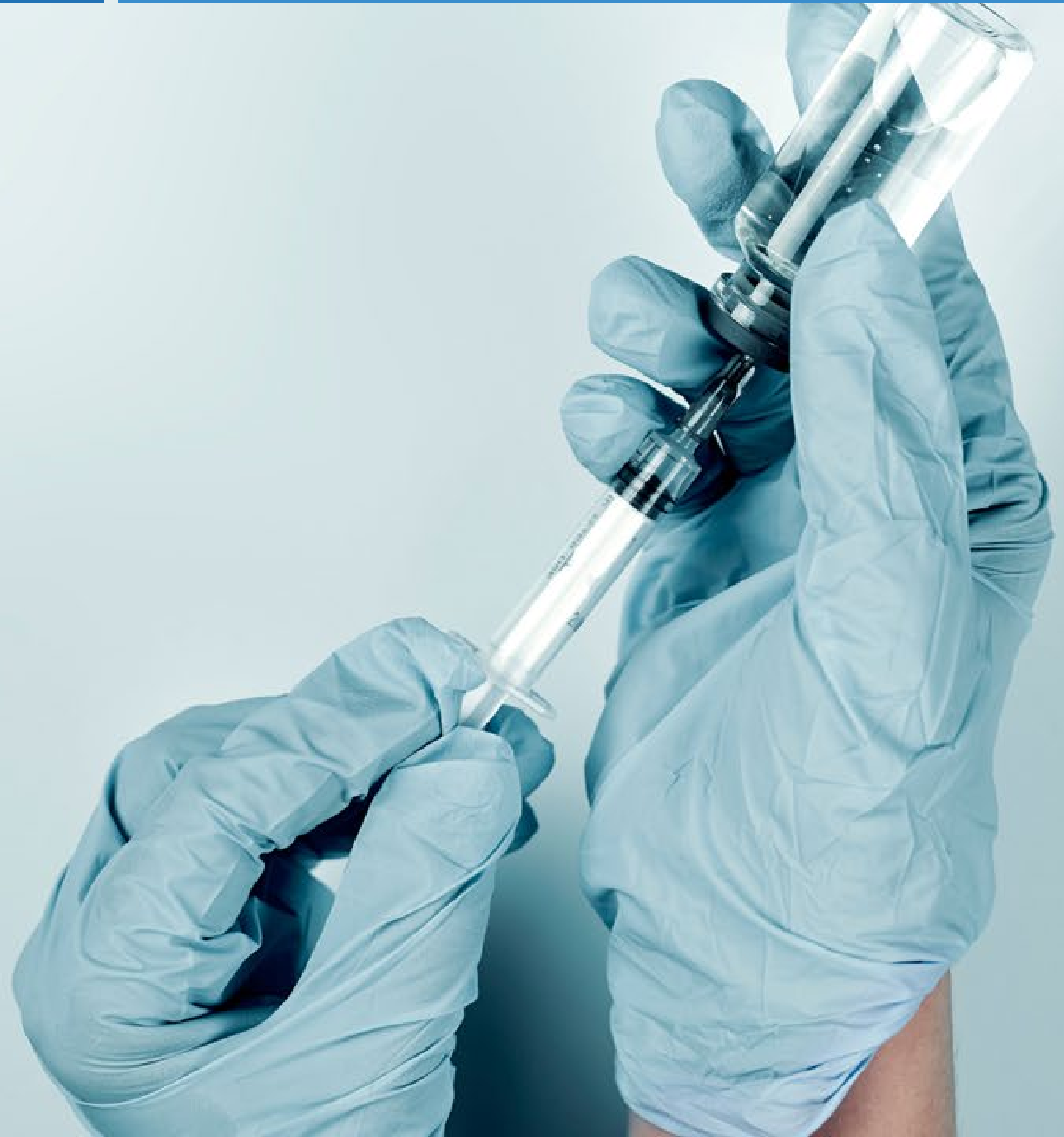

- Ministry of Health Uganda
- World Health Organization

  https://www.who.int/emergencies/diseases/novel-coronavirus-2019/covid-19-vaccines/explainers

- USAID Social and Behavior Change Activity Vaccination Toolkit
- San Francisco Department of Public Health
  - https://www.sfdph.org/dph/files/ig/vaccine/vaccine-ambassador-training-pdf.pdf

Acknowledgment

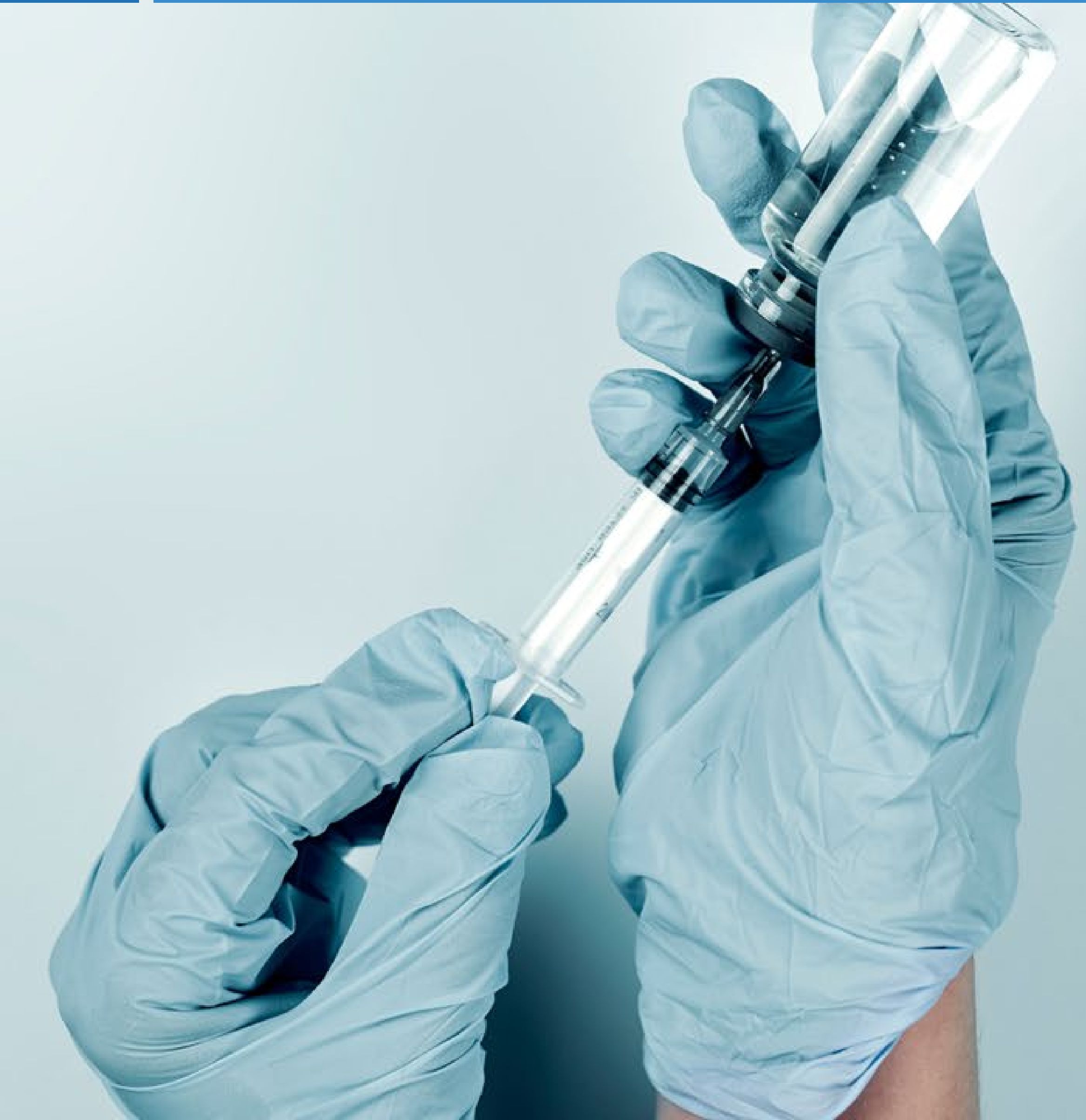

Funding: United States Centres for Disease Prevention and Control (CDC) grant (GH000045) awarded to Infectious Diseases Institute, Makerere University
